# Efficacy and Safety of Intravenous Thrombolysis in the Extended Time Window for Acute Ischemic Stroke: A Systematic Review and Meta-Analysis

**DOI:** 10.3390/jcm14155474

**Published:** 2025-08-04

**Authors:** Lina Palaiodimou, Nikolaos M. Papageorgiou, Apostolos Safouris, Aikaterini Theodorou, Eleni Bakola, Maria Chondrogianni, Georgia Papagiannopoulou, Odysseas Kargiotis, Klearchos Psychogios, Eftihia Polyzogopoulou, Georgios Magoufis, Georgios Velonakis, Jobst Rudolf, Panayiotis Mitsias, Georgios Tsivgoulis

**Affiliations:** 1 Second Department of Neurology, “Attikon” University Hospital, School of Medicine, National & Kapodistrian University of Athens, Rimini 1, Chaidari, 12462 Athens, Greece; 2 Stroke Unit, Metropolitan Hospital, 18547 Piraeus, Greece; 3 Department of Neurology, University General Hospital of Patras, University of Patras, 26504 Rio, Greece; 4 Emergency Medicine Clinic, “Attikon” University Hospital, School of Medicine, National & Kapodistrian University of Athens, 12462 Athens, Greece; 5 Interventional Radiology Unit, Second Department of Radiology, “Attikon” University Hospital, School of Medicine, National and Kapodistrian University of Athens, 12462 Athens, Greece; 6 Interventional Neuroradiology Unit, Metropolitan Hospital, 18547 Piraeus, Greece; 7 Second Department of Radiology, “Attikon” University Hospital, School of Medicine, National and Kapodistrian University of Athens, 12462 Athens, Greece; 8 Department of Neurology, Papageorgiou General Hospital, 56429 Thessaloniki, Greece; 9 Department of Neurology, University General Hospital of Heraklion, School of Medicine, University of Crete, 71500 Heraklion, Greece

**Keywords:** intravenous thrombolysis, acute ischemic stroke, extended-time window, functional outcome, symptomatic intracranial hemorrhage, meta-analysis

## Abstract

**Background/Objectives:** While intravenous thrombolysis (IVT) is the standard treatment for acute ischemic stroke (AIS) within 4.5 h of symptom onset, many patients present beyond this time window. Recent trials suggest that IVT may be both effective and safe in selected patients treated after the standard time window. **Methods:** We searched MEDLINE, Scopus, and ClinicalTrials.gov for randomized-controlled clinical trials (RCTs) and individual patient-data meta-analyses (IPDMs) of RCTs comparing IVT plus best medical treatment (BMT) to BMT alone in AIS patients who were last-known-well more than 4.5 h earlier. The primary efficacy outcome was a 90-day excellent functional outcome [modified Rankin Scale (mRS)-scores of 0–1]. Secondary efficacy outcomes included good functional outcome (mRS-scores 0–2) and reduced disability (≥1-point reduction across all mRS-strata). The primary safety outcome was symptomatic intracranial hemorrhage (sICH); secondary safety outcomes were any ICH and 3-month all-cause mortality. Subgroup analyses were performed stratified by different thrombolytics, time-windows, imaging modalities, and affected circulation. **Results:** Nine studies were included, comprising 1660 patients in the IVT-group and 1626 patients in the control-group. IVT significantly improved excellent functional outcome (RR = 1.24; 95%CI:1.14–1.34; I^2^ = 0%) and good functional outcome (RR = 1.18; 95%CI:1.05–1.33; I^2^ = 70%). IVT was associated with increased odds of reduced disability (common OR = 1.3; 95%CI:1.15–1.46; I^2^ = 0%) and increased risk of sICH (RR = 2.75; 95%CI:1.49–5.05; I^2^ = 0%). The rates of any ICH and all-cause mortality were similar between the two groups. No significant subgroup differences were documented. **Conclusions:** IVT in the extended time window improved functional outcomes without increasing mortality, despite a higher rate of sICH.

## 1. Introduction

Acute ischemic stroke (AIS) remains a leading cause of long-term disability and mortality worldwide [1]. In addition to its clinical burden, stroke imposes a substantial economic impact, accounting for billions in direct medical expenses and indirect costs such as lost productivity and long-term care [2]. Timely reperfusion with intravenous thrombolysis (IVT), using alteplase (tPA) or tenecteplase (TNK), is currently recommended by international guidelines for AIS patients presenting within 4.5 h from symptom onset or last known well [3,4,5]. More recently, a systematic review and meta-analysis provided conclusive evidence that TNK is in fact superior to tPA in this standard time window [6].

However, a substantial proportion of AIS patients present outside the standard time window, including those with wake-up strokes or unclear symptom onset [7]. Real-world data suggest that up to two-thirds of patients arrive too late to be considered for standard IVT [8,9,10,11]. These patients have traditionally been excluded from IVT due to uncertainty around timing, despite evidence that some may still have salvageable brain tissue based on imaging profiles. The use of advanced neuroimaging techniques—such as CT perfusion (CTP) or diffusion-weighted MRI (DWI MRI)—has allowed for more individualized treatment decisions by identifying patients with favorable perfusion patterns or limited core infarcts [12,13,14]. For those selected patients presenting between 4.5 and 9 h from last known well, or with strokes of unknown time of onset, including wake-up strokes, the use of IVT with tPA may also be considered when salvageable brain tissue is identified with the use of advanced imaging techniques, such as perfusion CT or diffusion-weighted MRI [3,5,15]. However, access to these imaging modalities is often limited in real-world settings, particularly in community hospitals and low-resource settings [12].

A recent systematic review and meta-analysis of randomized-controlled clinical trials (RCTs) has shown that TNK is effective and safe in the treatment of AIS for patients presenting beyond 4.5 h since they were last known well [16]. However, this systematic review was limited by the inclusion of RCTs with quite heterogeneous inclusion criteria and imaging protocols for selection. Since then, additional RCTs evaluating the efficacy and safety of IVT in patients presenting beyond the 4.5-h time window have been published or announced their results; however, the evidence remains heterogeneous [17,18,19]. Clinical consensus on expanding the thrombolysis window remains limited, in part due to these inconsistencies in trial design and patient selection.

In this context, we conducted a systematic review and aggregate-data meta-analysis to evaluate the efficacy and safety of IVT together with best medical therapy (BMT) compared to BMT alone in adult patients with AIS presenting beyond 4.5 h since they were last known well. By integrating data from the most recent high-quality trials, this study aims to assess the potential extension of the 4.5-h time window for IVT.

## 2. Materials and Methods

### 2.1. Standard Protocol Approvals, Registrations, and Patient Consents

The protocol for this study was registered in the International Prospective Register of Ongoing Systematic Reviews PROSPERO (registration ID: CRD420251072877; date of registration: 12 June 2025). This meta-analysis was conducted in accordance with the updated Preferred Reporting Items for Systematic Reviews and Meta-Analyses (PRISMA) guidelines [20]. Given the nature of this work as a systematic review and meta-analysis, neither ethics committee approval nor informed consent was required. No direct patient enrollment was involved; all data were derived from the studies included in this review.

### 2.2. Data Sources, Searches, and Study Selection

A comprehensive literature search was performed based on the Patient, Intervention, Comparison, and Outcome (PICO) framework [21] to identify relevant RCTs or individual patient-data meta-analyses (IPDM) of RCTs including adult patients with AIS presenting beyond 4.5 h of last known well (P: population) receiving IVT together with BMT (I: intervention) versus BMT alone with or without placebo (C: control) and investigating the outcomes of interest as outlined below (O: outcome). The “extended time window” in this context referred to patients presenting more than 4.5 h after stroke onset or last known well, including those with wake-up strokes or strokes of unknown onset. The thrombolytic agents in the IVT group included tPA or TNK. BMT was defined as standard care for patients with AIS according to the international guidelines, excluding IVT or other experimental agents.

We excluded observational cohorts, uncontrolled studies, case series, and case reports. Trials directly comparing two thrombolytic agents were also omitted from this systematic review and meta-analysis. Additionally, commentaries, editorials, and narrative reviews were not considered for inclusion.

Four reviewers (LP, NMP, AS, and AT) independently conducted the literature search. We queried MEDLINE, Scopus, and ClinicalTrials.gov using predefined terms such as “stroke”, “intravenous thrombolysis”, and “extended time window”. The full search strategy is detailed in the Supplement (eMethods). No restrictions were placed on language. The search covered all records available up to 15 June 2025. Additionally, reference lists of included studies and abstracts from international conferences were manually reviewed to ensure thorough coverage. Screening and selection of eligible studies were independently carried out by four reviewers (LP, NMP, EB, and GP), with any discrepancies resolved through discussion with a fifth adjudicator (GT).

### 2.3. Quality Control, Bias Assessment, and Data Extraction

Methodological quality and risk of bias were independently evaluated by four reviewers (LP, NMP, OK, KP) using the Cochrane Risk of Bias 2 (RoB 2) tool for RCTs [22]. Any discrepancies were resolved through discussion and consensus with a fifth reviewer (GT).

Data were extracted using a structured format, capturing key details such as study name, country, recruitment timeframe, characteristics of the intervention and control groups, number and baseline demographics of participants (e.g., age, sex, baseline NIHSS score, time from last known well), imaging criteria used for inclusion, and reported outcomes of interest.

### 2.4. Outcomes

The main efficacy endpoint was defined as an excellent functional outcome at 3 months, corresponding to a modified Rankin Scale (mRS) score of 0 or 1 [23]. Secondary efficacy outcomes included good functional outcome (mRS score of 0–2) [23] and reduced disability at 3 months, measured as a minimum 1-point improvement across the full mRS range.

The primary safety outcome was symptomatic intracranial hemorrhage (sICH), as reported in each individual study. Secondary safety outcomes included any intracranial hemorrhage (aICH) and overall mortality at 3 months.

### 2.5. Statistical Analysis

In the pairwise meta-analysis, we estimated risk ratios (RRs) with 95% confidence intervals (CIs) to compare dichotomous outcomes between patients treated with IVT plus BMT and those receiving BMT alone. For each outcome, pooled proportions with corresponding 95% CIs were determined for both treatment arms, utilizing the variance-stabilizing double arcsine transformation. To assess reduced disability at 3 months, we calculated the unadjusted common odds ratio (cOR) with 95% CI using a generic inverse variance method. Prespecified subgroup analysis was conducted stratified by the thrombolytic agent used (tPA vs. TNK), time frame as defined by each study (4.5–9 h, 4.5–24 h, >4.5 h with no upper limit, and wake-up stroke), use of imaging (advanced imaging, including CTP or DWI MRI vs. standard imaging, including non-contrast CT), and affected circulation (anterior, posterior, or both) for the primary efficacy and safety outcome. A sensitivity analysis for the primary efficacy and safety outcome was also performed, excluding studies in which the patients could receive endovascular treatment (EVT). All outcomes were assessed based on intention-to-treat analysis. For the primary efficacy outcome, the number needed to treat (NNT) was calculated using the formula: NNT = $\frac{1}{\left(RR-1\right)\;x\;rate\;in\;BMT\;group}$ as previously described [24]. Similarly, for the primary safety outcome, the number needed to harm (NNH) was calculated using the formula: NNH = $\frac{1}{\left(RR-1\right)\;x\;rate\;in\;BMT\;group}$. To evaluate the balance in baseline characteristics between treatment groups, we used odds ratios for categorical variables and standardized mean differences for continuous variables. In cases where continuous data were reported as medians with interquartile ranges, we approximated the mean and standard deviation using a quantile-based estimation approach [25]. Pooled effect estimates were calculated using a random-effects model, as described by DerSimonian and Laird [26].

Statistical significance was defined as a two-sided *p*-value less than 0.05. Heterogeneity among studies was evaluated using both the I^2^ statistic and Cochran’s Q test. For interpretation, I^2^ values below 25% were considered low, 25–50% moderate, and values above 50% indicative of substantial heterogeneity. A *p*-value of less than 0.1 was used as the threshold for significance in the Q test. To assess potential small-study effects, which may suggest publication bias, we applied funnel plot analysis and Egger’s linear regression test when at least four studies were available for a given outcome [27]. All statistical analyses were conducted using R software (version 3.5.0) with the ‘meta’ package [28].

## 3. Results

### 3.1. Literature Search and Included Studies

**Figure 1:**
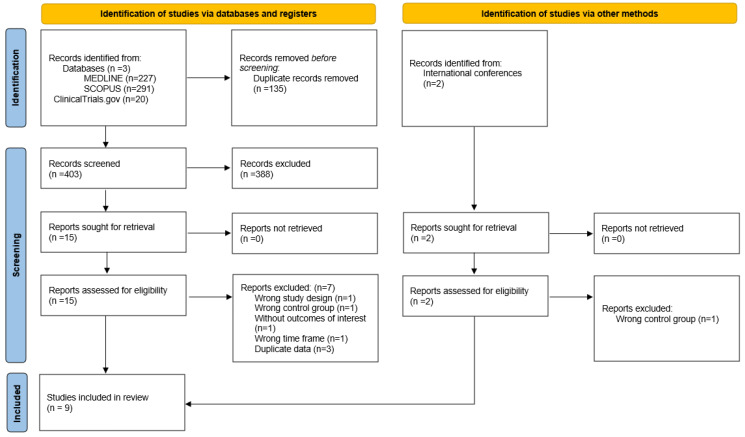
Flow chart of the systematic review.

The systematic database search yielded a total of 227, 291, and 20 records from the MEDLINE, SCOPUS, and ClinicalTrials.gov databases, respectively (Figure 1). After excluding duplicates and initial screening, we retrieved the full text of 15 records that were considered potentially eligible for inclusion. After reading the full-text articles, 7 were further excluded (Table S1) [29,30,31,32,33,34,35]. Furthermore, after searching the reference lists of published articles and international conference abstracts, 2 additional records were considered potentially eligible [36,37], with 1 of them finally included. Finally, we identified 8 eligible RCTs and 1 IPDM of RCTs (pooling data from 3 RCTs: EXTEND [38], ECASS4-EXTEND [39], and EPITHET [40]) for inclusion in the systematic review and meta-analysis (Table 1) [17,18,19,37,41,42,43,44,45]. This systematic review and meta-analysis included a total of 3286 patients with AIS that were presented beyond 4.5 h from the time they were last known well, receiving either IVT together with BMT [*n* = 1660; mean age 69 years; 39% female; mean baseline National Institutes of Health Stroke Scale (NIHSS) score of 9.7; presenting 11 h since they were last known well] versus BMT alone (*n* = 1626; mean age 69.4 years; 41% female; mean baseline NIHSS score of 9.3; presenting 11.5 h since they were last known well; Figures S1–S4). There were no significant baseline differences in the two groups. Regarding imaging in the included studies, strategies varied according to trial design. The EXPECTS [19] and TWIST [43] trials used non-contrast CT to exclude ICH prior to randomization, without the use of advanced imaging. The ECASS-4-EXTEND [39], EPITHET [40], ROSE-TNK [42,45], THAWS [42], and WAKE-UP [44] used MRI for patient selection, requiring a DWI-FLAIR mismatch greater than 20% for inclusion. The EXTEND [38], TIMELESS [17], and TRACE III [18] trials allowed for either MRI or CTP to identify the ischemic penumbra prior to randomization. The HOPE trial [37] exclusively used CTP for patient selection.

### 3.2. Quality Control of Included Studies

The risk of bias assessment for the included RCTs is summarized in Figure S5. All studies employed an appropriate randomization process, resulting in a low risk of bias for that domain. The three trials included in the IPDM of RCTs by Campbell et al. [41] and the TIMELESS [17] trial used placebo in the control group and did not present deviations from the intended intervention, leading to low risk of bias in this domain. The WAKE-UP [44] trial also used a placebo but excluded 79 patients after randomization, and the remaining trials presented some deviations from the intended intervention, resulting in some concerns for this domain [18,19,37,42,43,45]. The EXPECTS [19], WAKE-UP [44], and the IPDM of RCTs by Campbell et al. [41] reported some missing outcome data; however, the proportion of missing data did not exceed 10% of the enrolled population, resulting in low risk of bias in this domain. The remaining trials did not report any missing outcome data. All studies had a low risk of bias in the measurement of outcomes, as outcome assessors were blinded to treatment allocation. Additionally, the risk of bias due to selective reporting was judged to be low across all included studies.

### 3.3. Quantitative Analyses

An overview of the analyses for primary and secondary outcomes is presented in Table 2.

**Figure 2:**
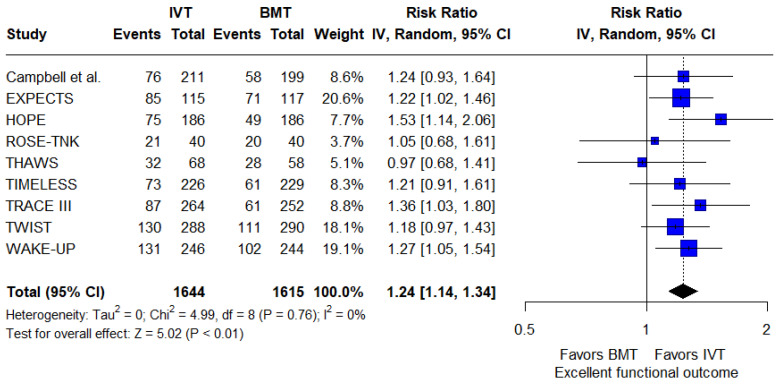
Forest plot presenting the risk ratio of excellent functional outcome at 3 months among patients receiving intravenous thrombolysis (IVT) plus best medical treatment (BMT) versus BMT alone. Blue squares indicate study-specific estimates (size reflects study weight), with horizontal black lines showing 95% confidence intervals. The black diamond represents the pooled estimate, and the black dashed line marks the summary risk ratio. The black vertical line at 1.0 indicates no effect.

Regarding the primary efficacy outcome, patients receiving IVT had significantly higher rates of achieving an excellent functional outcome at 3 months compared to those receiving BMT alone (RR: 1.24; 95%CI: 1.14–1.34; *p* < 0.001; 9 studies; I^2^ = 0%; *p* for Cochran’s Q = 0.76; Figure 2). The NNT was 12 (95%CI: 9–21). There were no significant subgroup differences based on the thrombolytic agent used (*p* for subgroup differences = 0.72), the time window applied (*p* for subgroup differences = 0.88), the neuroimaging method used (*p* for subgroup differences = 0.58), or the affected territory (*p* for subgroup differences = 0.93; Figure S6).

**Figure 3:**
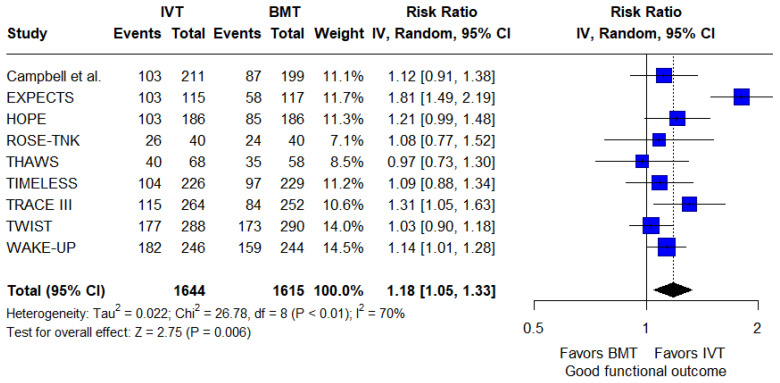
Forest plot presenting the risk ratio of good functional outcome at 3 months among patients receiving intravenous thrombolysis (IVT) plus best medical treatment (BMT) versus BMT alone. Blue squares indicate study-specific estimates (size reflects study weight), with horizontal black lines showing 95% confidence intervals. The black diamond represents the pooled estimate, and the black dashed line marks the summary risk ratio. The black vertical line at 1.0 indicates no effect.

**Figure 4:**
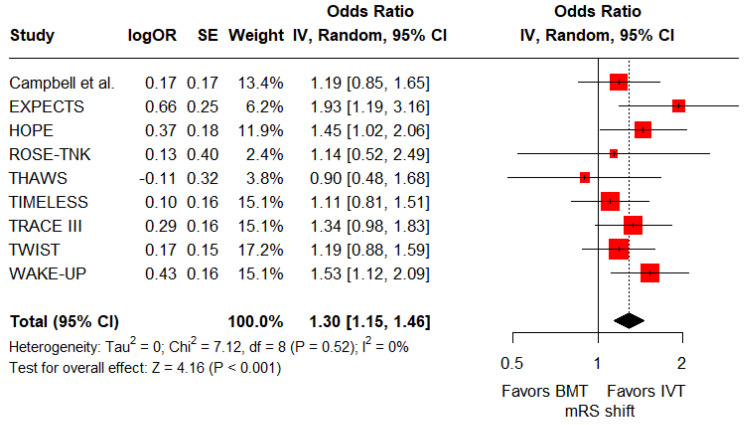
Forest plot presenting the common odds ratio of reduced disability at 3 months among patients receiving intravenous thrombolysis (IVT) plus best medical treatment (BMT) versus BMT alone. Red squares indicate study-specific estimates (size reflects study weight), with horizontal black lines showing 95% confidence intervals. The black diamond represents the pooled estimate, and the black dashed line marks the summary odds ratio. The black vertical line at 1.0 indicates no effect.

Concerning secondary efficacy outcomes, the rates of good functional outcome were significantly higher for patients receiving IVT in addition to BMT compared to those receiving BMT alone (RR: 1.18; 95%CI: 1.05–1.33; *p* = 0.006; 9 studies; I^2^ = 70%; *p* for Cochran’s Q < 0.01; Figure 3). Furthermore, patients receiving IVT had significantly higher odds of reduced disability at 3 months compared to those receiving BMT alone (common OR: 1.3; 95%CI: 1.15–1.46; *p* < 0.001; 9 studies; I^2^ = 0%; *p* for Cochran’s Q = 0.52; Figure 4).

**Figure 5:**
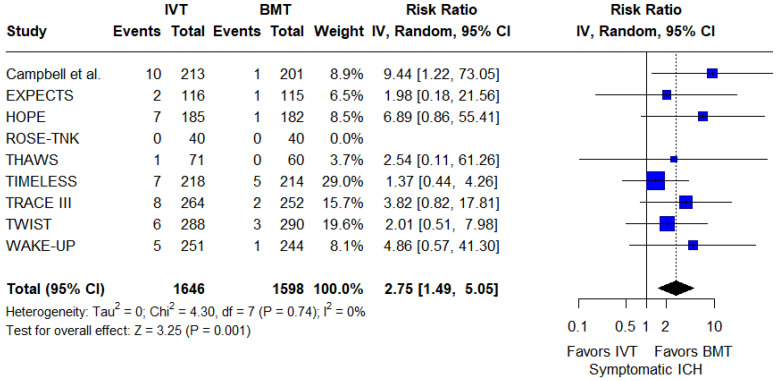
Forest plot presenting the risk ratio of symptomatic intracranial hemorrhage among patients receiving intravenous thrombolysis (IVT) plus best medical treatment (BMT) versus BMT alone. Blue squares indicate study-specific estimates (size reflects study weight), with horizontal black lines showing 95% confidence intervals. The black diamond represents the pooled estimate, and the black dashed line marks the summary risk ratio. The black vertical line at 1.0 indicates no effect.

Regarding the primary safety outcome, the rate of sICH was significantly higher among patients treated with IVT (RR: 2.75; 95%CI: 1.49–5.05; *p* = 0.001; 9 studies; I^2^ = 0%; *p* for Cochran’s Q = 0.74; Figure 5). The NNH was 53 (95%CI: 29–233). Subgroup analyses did not reveal significant differences based on the thrombolytic agent used (*p* for subgroup differences = 0.16), the time window applied (*p* for subgroup differences = 0.59), the neuroimaging method (*p* for subgroup differences = 0.55), or the affected circulation (*p* for subgroup differences = 0.93; Figure S7).

**Figure 6:**
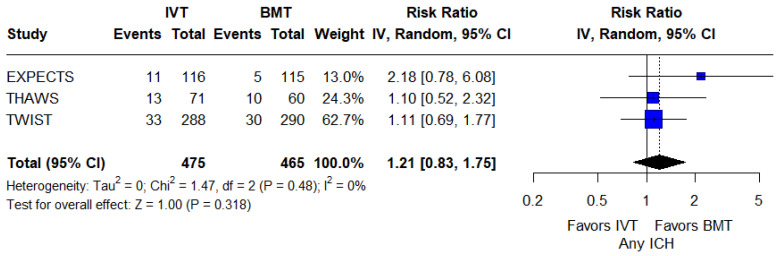
Forest plot presenting the risk ratio of any intracranial hemorrhage among patients receiving intravenous thrombolysis (IVT) plus best medical treatment (BMT) versus BMT alone. Blue squares indicate study-specific estimates (size reflects study weight), with horizontal black lines showing 95% confidence intervals. The black diamond represents the pooled estimate, and the black dashed line marks the summary risk ratio. The black vertical line at 1.0 indicates no effect.

**Figure 7:**
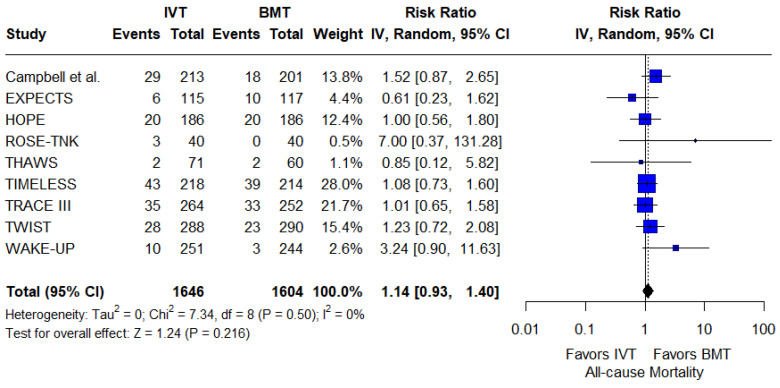
Forest plot presenting the risk ratio of 3-month all-cause mortality among patients receiving intravenous thrombolysis (IVT) plus best medical treatment (BMT) versus BMT alone. Blue squares indicate study-specific estimates (size reflects study weight), with horizontal black lines showing 95% confidence intervals. The black diamond represents the pooled estimate, and the black dashed line marks the summary risk ratio. The black vertical line at 1.0 indicates no effect.

For secondary safety outcomes, the rates of any ICH were similar between groups (RR: 1.21; 95%CI: 0.83–1.75; *p* = 0.32; 3 studies; I^2^ = 0%; *p* for Cochran’s Q = 0.48; Figure 6). Similarly, all-cause mortality at 3 months did not differ between the two groups (RR: 1.14; 95%CI: 0.93–1.40; *p* = 0.22; 9 studies; I^2^ = 0%; *p* for Cochran’s Q = 0.50; Figure 7).

In the sensitivity analysis, after excluding studies in which patients could receive EVT, the results remained consistent. Patients treated with IVT showed significantly higher rates of excellent functional outcome at 3 months compared to those receiving BMT alone (RR: 1.25; 95%CI: 1.14–1.38; *p* < 0.001; 7 studies; I^2^ = 0%; *p* for Cochran’s Q = 0.59; Figure S8). Similarly, the rate of sICH remained significantly higher in the IVT group (RR: 4.57; 95% CI: 1.95–10.7; *p* < 0.001; 7 studies; I^2^ = 0%; *p* for Cochran’s Q = 0.94; Figure S9).

The pooled proportions per arm for each outcome of interest are presented in Table S2 in the Supplement.

Assessment of publication bias using funnel plot inspection revealed no evidence of asymmetry for any of the outcomes, except for the outcome of good functional outcome at 90 days (Figures S10–S14). For this outcome, visual asymmetry was observed due to the EXPECTS [19] study, without evidence of small study effects (*p* for Egger’s test= 0.73).

## 4. Discussion

In this systematic review and meta-analysis of eight RCTs and one IPDM of RCTs comprising 3286 patients with AIS presenting beyond the standard 4.5-h time window since they were last known well, we found that IVT was associated with a significantly higher likelihood of achieving excellent functional outcome (NNT = 12), good functional outcome, and higher odds of reduced disability at 90 days. Although IVT was linked to an increased risk of sICH (NNH = 53), it was not associated with a significantly higher risk of any ICH or all-cause mortality at 3 months. These findings support the therapeutic potential of IVT in patients treated beyond the conventional 4.5-h time window.

Our primary efficacy analysis suggests that IVT beyond the conventional 4.5-h window is associated with a significant improvement in functional outcomes among patients with AIS, with an NNT of 12 for achieving excellent functional outcome. IVT was also associated with higher rates of good functional outcome and reduced overall disability at 3 months, as evidenced by the favorable shift across the entire mRS spectrum. Particularly in clinical situations where patients present late—such as wake-up strokes or strokes of unknown onset—these results highlight the opportunity for offering IVT to additional patients who may still harbor salvageable brain tissue, thereby improving outcomes and reducing long-term disability.

Regarding safety outcomes and treatment complications, our analysis suggested that the use of IVT beyond 4.5 h was associated with a significantly increased risk of sICH, consistent with the known risks of thrombolytic therapy. The pooled proportion of sICH was calculated at 3% for the IVT arm, which is consistent with the one reported for IVT in the standard time window [46,47,48]. Moreover, the NNH for sICH was calculated at 53, suggesting a net clinical benefit favoring IVT when accounting for the NNT of 12 for excellent functional outcome in those patients. Importantly, the increase in sICH did not result in a corresponding increase in all-cause mortality at 3 months. These findings underscore that while IVT carries inherent hemorrhagic risk, its use in extended time windows may still be justified given the absence of increased mortality and the potential for improved functional outcomes. Finally, the rate of any ICH was similar between the groups, supporting the overall acceptable safety profile of IVT in the extended time window.

Subgroup analyses demonstrated consistent efficacy of IVT across a wide range of predetermined subgroup analyses, including different thrombolytics, time windows, clinical presentations, and imaging approaches. Both tPA and TNK were associated with improved functional outcomes, suggesting that either thrombolytic agent can be effectively used in the extended time window. TNK offers practical advantages over tPA, including single-bolus administration, longer half-life, and quicker delivery [49]; thus, it may be preferred in some clinical settings [50,51,52]. Interestingly, the CHABLIS-T II [29] and ETERNAL-LVO [36] trials confirmed similar rates of excellent functional outcome or good functional outcome between TNK-treated patients and controls, among whom some patients received tPA. Similarly, the rates of sICH or 90-day all-cause mortality did not differ significantly between the two groups. More RCTs evaluating TNK in the extended time window are ongoing [POST-ETERNAL (NCT05105633), RESILIENT TNK (NCT05199662)], aiming to further delineate this issue.

The benefit of IVT was observed regardless of the specific time interval (4.5 to 9 h, up to 24 h, or in wake-up strokes), indicating broad applicability among late-presenting AIS patients. Similarly, IVT was effective in both anterior and posterior circulation strokes. Notably, the use of advanced neuroimaging (CTP or DWI MRI) versus standard imaging (non-contrast CT) did not significantly modify the treatment effect, supporting the feasibility of IVT even in settings without access to advanced imaging. However, it is important to note that most included studies were conducted in high-income countries with well-established stroke care systems. The generalizability of our findings to low- and middle-income countries, where access to advanced imaging, thrombolytic agents, and organized stroke units may be limited, remains uncertain and warrants further investigation. For the primary safety outcome (sICH), no significant subgroup differences were observed based on thrombolytic agent, imaging modality, vascular territory, or time window, indicating that the elevated risk of hemorrhage associated with IVT appears consistent across different clinical contexts.

The sensitivity analysis, which excluded studies where patients could receive EVT, confirmed the robustness of our findings. Even after removing these studies, IVT remained significantly associated with improved excellent functional outcomes at 3 months, while the increased risk of sICH persisted. These results suggest that the benefits and risks of IVT beyond 4.5 h are consistent regardless of whether EVT is part of the treatment strategy, reinforcing the potential value of extending thrombolysis in diverse clinical settings. Currently, bridging reperfusion therapy, combining IVT and EVT, is recommended by international guidelines for the standard time window, where EVT alone was shown not to be non-inferior compared to bridging therapy [53]. More recently, the BRIDGE-TNK [54] trial has shown that functional independence at 90 days was higher with intravenous TNK plus EVT than with EVT alone again in the standard time window of 4.5 h. It remains to be evaluated whether the combination of treatments should be pursued in the extended time windows as well.

Funnel plot inspection revealed asymmetry for the outcome of mRS 0–2 at 90 days, driven by the EXPECTS study [19], which produced notable heterogeneity. This study reported a high proportion of patients achieving mRS 0–2 compared to the rest of the included studies. However, its patient population had a relatively low baseline stroke severity, with a mean NIHSS of 4.8. To assess the impact of this heterogeneity, we conducted an additional sensitivity analysis excluding the EXPECTS study for the primary efficacy outcome and the good functional outcome, which demonstrated that the overall results remained consistent and robust, confirming that the inclusion of this study did not materially influence our main findings (Figure S15).

Several ongoing RCTs are expected to provide further clarity on the role of IVT in extended time windows and specific patient subgroups. The TNK-MeVO (NCT06559436), being conducted in China, is evaluating TNK versus BMT in patients with AIS due to distal medium vessel occlusion, a population that remains underrepresented in existing studies. Similarly, the OPTION trial (NCT05752916) is assessing TNK versus BMT in AIS patients selected using CTP to identify salvageable tissue. These studies will help refine patient selection and inform the future role of IVT beyond standard time limits. As these data mature, they may help identify patient subgroups most likely to benefit from IVT. Upcoming secondary analyses from these trials may further elucidate key factors such as optimal thrombolytic choice, imaging-based selection criteria, and the influence of time from last known well on treatment efficacy and safety.

Our results align with those from recent meta-analyses, reinforcing the efficacy of IVT beyond 4.5 h [55,56]. However, our analysis provides notable improvements. Unlike Günkan et al. [55], we did not exclude studies involving EVT and avoided the inclusion of EXIT-BT [35], where an investigational neuroprotective agent was administered in both arms—potentially diluting the true effect of IVT. Compared to the meta-analysis of Al-Janabi et al. [56], we included the EXPECTS [19] trial, which focuses exclusively on posterior circulation strokes. We also incorporated the HOPE trial [37] that was recently announced at the International Stroke Congress in 2025, and it was not included in any of the previous meta-analyses. Importantly, our work included a detailed set of subgroup analyses across different thrombolytic agents, time windows, imaging modalities, and vascular territories, offering a more detailed understanding of treatment effects. By including the most up-to-date and comprehensive evidence base, our analysis offers a more complete and robust assessment of IVT in the extended time window.

Despite its strengths, there are also important limitations in this study to consider. As a study-level analysis, it incorporates data from trials with varying inclusion criteria, imaging protocols, and outcome definitions, which may introduce heterogeneity. The HOPE trial [37], although included, has not yet been published, and data were obtained from its presentation at the 2025 International Stroke Congress, which limits the availability of detailed methodology and results. Some heterogeneity was noted in the mRS 0–2 outcome; however, sensitivity analyses excluding the EXPECTS trial supported the consistency of our findings. Although we performed extensive subgroup analyses to explore potential effect modifiers, such analyses based on aggregate data are inherently limited and may not reflect patient-level variability. Additionally, prehospital factors (emergency medical system activation, transport delays, and onset-to-door time) as well as detailed in-hospital workflow metrics such as door-to-CT time and imaging-to-treatment intervals could not be assessed, as these were not consistently reported across the included trials. Future IPDMs will be essential to guide optimal patient selection and provide definitive answers regarding IVT in extended time windows.

## 5. Conclusions

In conclusion, this meta-analysis suggests that IVT is an effective treatment for AIS beyond the conventional 4.5-h window, leading to improved rates of excellent and good functional outcomes. These results are further supported by the documented association between IVT and increased odds of reduced disability across all mRS-strata. While an increased risk of sICH was observed, this did not translate into higher mortality, supporting the overall safety of treatment in the extended window. As additional trial data become available, IVT may be increasingly adopted as a standard therapeutic option for AIS patients presenting beyond 4.5 h from symptom onset.

## Figures and Tables

**Table 1:** Baseline characteristics of studies included in this systematic review and meta-analysis.

Study		Country	Recruitment Period	Imaging	Inclusion	Drug	Group	Age	Female	NIHSS	EVT	LKW
Campbell et al. (IPDM) [41]	ECASS4-EXTEND [39]	Europe	Jan 2014–Sep 2017	MRI	onset 4.5–9 h NIHSS 4–26 Perfusion volume > 20 mL, mismatch > 20%	Alteplase 0.9 mg/kg (maximum dose 90 mg)	IVT (n = 213)	73.2 ± 12.2	94	12 (7–17)	-	471 (346–485) minutes
	EPITHET [40]	Australia, New Zealand Belgium UK	Apr 2001–Jan 2007	MRI	onset 3–6 h NIHSS > 4 mRS < 3 Perfusion volume > 10 mL, mismatch > 20%							
	EXTEND [38]	Australia, New Zealand, Taiwan, Finland	Aug 2010–Jun 2018	CTP, MRI	onset 4.5–9 h NIHSS 4–26 mRS < 2 Perfusion volume > 10 mL, mismatch > 20%, ischemic core < 70 mL		PCB (n = 201)	72 ± 12.3	85	10 (6–16)	-	413 (353–480) minutes
EXPECTS [19]		China	Aug 2022–May 2024	CT	onset 4.5–24 h, NIHSS > 0, mRS < 2, PC-ASPECTS > 6	Alteplase 0.9 mg/kg (maximum dose 90 mg)	IVT (n = 117)	64 (57–76)	42	3 (2–6)	-	NA
							BMT (n = 117)	63 (55–74)	39	3 (1–6)	-	NA
HOPE [37]		China		CTP	onset 4.5–24 h, NIHSS 4–26, Ischemic core volume < 70 mL, penumbra volume > 10 mL, mismatch > 20%, mRS < 2	Alteplase 0.9 mg/kg (maximum dose 90 mg)	IVT (n = 186)	72 (62–80)	84	10 (6–15)	-	NA
							BMT (n = 186)	73 (65–80)	76	10 (6–14)	-	NA
ROSE-TNK [45]		China	Mar 2021–Jul 2022	MRI	onset 4.5–24 h, NIHSS 6–25, infarct < 1/3 of MCA, < 1/2 of ACA, < 1/2 of PCA, ischemic core volume < 70 mL and DWI—FLAIR mismatch, mRS < 2	Tenecteplase 0.25 mg/kg (maximum dose 25 mg)	IVT (n = 40)	62.7 ± 8.9	9	7.5 (6–10.75)	-	NA
							BMT (n = 40)	62.8 ± 5.6	14	7 (6–8.75)	-	NA
THAWS [42]		Japan	May 2014–Jul 2018	MRI	>4.5 h from symptoms onset, NIHSS 2–24, DWI—FLAIR MRI mismatch	Alteplase 0.6 mg/kg	IVT (n = 70)	73.2 ± 12.4	25	N/A	-	NA
							BMT (n = 61)	75.8 ± 11.90	30	N/A	-	NA
TIMELESS [17]		Canada, United States	Mar 2019–Dec 2022	CTP, MRI	onset 4.5–24 h, NIHSS > 5, occlusion of ICA, M1, M2 on CTA or MRA, Ischemic core volume < 70 mL, penumbra volume > 15 mL, ratio of ischemic tissue to the initial infarct volume > 1.8, mRS 0–2	Tenecteplase 0.25 mg/kg (maximum dose 25 mg)	IVT (n = 228)	72 (62–79)	122	12 (8–17)	176	12.7 (9.2–15.8) h
							PCB (n = 230)	73 (63–82)	123	12 (8–18)	178	13 (9–16.9) h
TRACE III [18]		China	Jan 2022–Nov 2023	CTP, MRI	onset 4.5–24 h, NIHSS 6–25, occlusion of ICA, M1, M2 on CTA or MRA, perfusion mismatch on CTP or DWI-MRI, Ischemic core volume < 70 mL, ratio of hypoperfused tissue to ischemic core volume > 1.8, penumbra volume > 15 mL, mRS < 2	Tenecteplase 0.25 mg/kg (maximum dose 25 mg)	IVT (n = 264)	67 (58–75)	81	11 (7–15)	-	12.4 (8.8–16.3) h
							BMT (n = 252)	68 (59–76)	85	10 (7–14)	-	12.8 (9–17.5) h
TWIST [43]		International	Jun 2017–Sep 2021	CT	onset upon awakening, NIHSS > 2 or aphasia	Tenecteplase 0.25 mg/kg (maximum dose 25 mg)	IVT (n = 288)	72.7 ± 11.3	124	6 (5–11)	18	NA
							BMT (n = 290)	72.9 ± 11.6	122	6 (5–10)	42	NA
WAKE-UP [44]		Europe	Sep 2012–Jun 2017	MRI	onset upon awakening or unknown onset but >4.5 h of last known well, DWI-FLAIR mismatch, NIHSS < 26, mRS < 2	Alteplase 0.9 mg/kg (maximum dose 90 mg)	IVT (n = 254)	65.3 ± 11.2	89	6 (4–9)	-	10.3 (8.1–12) h
							PCB (n = 249)	65.2 ± 11.9	89	6 (4–9)	-	10.4 (8.1–12.1) h

IPDM: Individual patent-data meta-analyses; NIHSS: National Institutes of Health Stroke Scale; IVT: intravenous thrombolysis; BMT: best medical treatment; EVT: Endovascular treatment; LKW: Last-known-well; ICA: internal carotid artery; M1: M1 segment of the middle cerebral artery; M2: M2 segment of the middle cerebral artery; PCA: posterior circulation artery; mRS: modified Rankin Scale; ASPECTS: Alberta Stroke Program Early CT Score; pc-ASPECTS: posterior circulation Alberta Stroke Program Early CT Score; NA: not applicable.

**Table 2:** Overview of analyses for the outcomes of interest.

Outcome	Effect Measure	Value (95% CI)	*p*-Value	N of Studies	I^2^ (P for Cochrane Q)	P for Subgroup Differences
**Primary Efficacy Outcome**						
Excellent Functional Outcome	RR, NNT	1.24 (1.14–1.34) 12 (9–21)	<0.001	9	0% (0.76)	0.72 (Thrombolytic Agent) 0.88 (Time) 0.58 (Neuroimaging) 0.93 (Affected Circulation)
**Secondary Efficacy Outcomes**						
Good Functional Outcome	RR	1.18 (1.05–1.33)	0.006	9	70% (<0.01)	-
Reduced Disability	cOR	1.30 (1.15–1.46)	<0.001	9	0% (0.52)	-
**Primary Safety Outcome**						
Symptomatic Intracranial Hemorrhage	RR, NNH	2.75 (1.49–5.05) 53 (29–233)	0.001	9	0% (0.74)	0.16 (Thrombolytic Agent) 0.59 (Time) 0.55 (Neuroimaging) 0.93 (Affected Circulation)
**Secondary Safety Outcome**						
Any Intracranial Hemorrhage	RR	1.21 (0.83–1.75)	0.318	3	0% (0.48)	-
All-Cause Mortality	RR	1.14 (0.93–1.40)	0.22	9	0% (0.50)	-

RR: risk ratio; NNT: number needed to treat; NNH: number needed to harm; cOR: common odds ratio; CI: confidence interval.

## Data Availability

All data generated or analyzed in this study are included in this article and its Supplementary Information files.

## Supplementary Materials

The following supporting information can be downloaded at: https://www.mdpi.com/article/10.3390/jcm14155474/s1, eMethods: Complete search algorithm used in MEDLINE (using PubMed); Complete search algorithm used in Scopus; Complete search algorithm used in ClinicalTrials.gov; Table S1. Excluded studies with reasons of exclusion; Table S2. Pooled proportion of outcomes of interest in each arm; eReferences; Figure S1. Forest plots presenting the mean age (in years) among patients treated with intravenous thrombolysis (IVT) together with best medical treatment (BMT; Panel A), the mean age (in years) among patients treated with BMT alone (Panel B), and the standardized mean difference of age (in years) among the patients treated with IVT together with BMT versus BMT alone (Panel C); Figure S2. Forest plots presenting the pooled proportion of female patients among those treated with IVT together with BMT (Panel A), the pooled proportion of female patients treated with BMT alone (Panel B), and the odds ratio of female patients treated with IVT together with BMT versus BMT (Panel C); Figure S3. Forest plots presenting the mean NIHSS among patients treated with IVT together with BMT (Panel A), the mean NIHSS among patients treated with BMT alone (Panel B), and the standardized mean difference of NIHSS among the patients treated with IVT together with BMT versus BMT alone (Panel C); Figure S4. Forest plots presenting the mean time since they were last know well (in hours) among patients treated with IVT together with BMT (Panel A), the mean time since they were last know well (in hours) among patients treated with BMT alone (Panel B), and the standardized mean difference of time (in hours) among the patients treated with IVT together with BMT versus BMT alone (Panel C); Figure S5. Traffic light plot (A) and summary plot (B) presenting the quality assessment of the included randomized-controlled clinical trials (RCTs) and the individual patient-data meta-analysis (IPDM) of RCTs, using the Cochrane Collaboration tool (RoB 2); Figure S6. Forest plot presenting the risk ratio of excellent functional outcome at 3 months among patients receiving IVT together with BMT versus BMT alone, stratified by thrombolytic agent used (Panel A), stratified by time of presentation (Panel B), stratified by neuroimaging used in each study (Panel C) and stratified by affected circulation (Panel D); Figure S7. Forest plot presenting the risk ratio of symptomatic intracranial hemorrhage (sICH) among patients receiving IVT together with BMT versus BMT alone, stratified by thrombolytic agent used (Panel A), stratified by time of presentation (Panel B), stratified by neuroimaging used in each study (Panel C) and stratified by affected circulation (Panel D); Figure S8. Forest plot presenting the risk ratio of excellent functional outcome among patients receiving IVT together with BMT versus BMT alone, after excluding studies where patients underwent endovascular treatment (sensitivity analysis); Figure S9. Forest plot presenting the risk ratio of symptomatic intracranial hemorrhage among patients receiving IVT together with BMT versus BMT alone, after excluding studies where patients underwent endovascular treatment (sensitivity analysis); Figure S10. Funnel plot on the reported rates of excellent functional outcome at 3 months (*p* for Egger’s test = 0.62); Figure S11. Funnel plot on the reported rates of good functional outcome at 3 months (*p* for Egger’s test = 0.73); Figure S12. Funnel plot on the reported odds of reduced disability at 3 months (*p* for Egger’s test = 0.93); Figure S13. Funnel plot on the reported rates of symptomatic intracranial hemorrhage (*p* for Egger’s test = 0.13); Figure S14. Funnel plot on the reported rates of all-cause mortality at 3 months (*p* for Egger’s test = 0.35); Figure S15. Forest plot presenting the risk ratio of excellent functional outcome among patients receiving IVT together with BMT versus BMT alone, after excluding EXPECTS study (Panel A) and the risk ratio of good functional outcome among patients receiving IVT together with BMT versus BMT alone, after excluding EXPECTS study (Panel B).

## References

[1] GBD 2021 Stroke Risk Factor Collaborators (2024). Global, regional, and national burden of stroke and its risk factors, 1990–2021: A systematic analysis for the Global Burden of Disease Study 2021. Lancet Neurol..

[2] Gerstl J.V.E., Blitz S.E., Qu Q.R., Yearley A.G., Lassarén P., Lindberg R., Gupta S., Kappel A.D., Vicenty-Padilla J.C., Gaude E. (2023). Global, Regional, and National Economic Consequences of Stroke. Stroke.

[3] Berge E., Whiteley W., Audebert H., De Marchis G.M., Fonseca A.C., Padiglioni C., de la Ossa N.P., Strbian D., Tsivgoulis G., Turc G. (2021). European Stroke Organisation (ESO) guidelines on intravenous thrombolysis for acute ischaemic stroke. Eur. Stroke J..

[4] Alamowitch S., Turc G., Palaiodimou L., Bivard A., Cameron A., De Marchis G.M., Fromm A., Kõrv J., Roaldsen M.B., Katsanos A.H. (2023). European Stroke Organisation (ESO) expedited recommendation on tenecteplase for acute ischaemic stroke. Eur. Stroke J..

[5] Powers W.J., Rabinstein A.A., Ackerson T., Adeoye O.M., Bambakidis N.C., Becker K., Biller J., Brown M., Demaerschalk B.M., Hoh B. (2019). Guidelines for the Early Management of Patients With Acute Ischemic Stroke: 2019 Update to the 2018 Guidelines for the Early Management of Acute Ischemic Stroke: A Guideline for Healthcare Professionals From the American Heart Association/American Stroke Association. Stroke.

[6] Palaiodimou L., Katsanos A.H., Turc G., Asimakopoulos A.G., Mavridis D., Schellinger P.D., Theodorou A., Lemmens R., Sacco S., Safouris A. (2024). Tenecteplase vs Alteplase in Acute Ischemic Stroke Within 4.5 Hours: A Systematic Review and Meta-Analysis of Randomized Trials. Neurology.

[7] Mansour M. (2023). Reperfusion Therapies in Acute Ischemic Stroke Beyond the Conventional Time Window: A Narrative Review. Cureus.

[8] Tong D., Reeves M.J., Hernandez A.F., Zhao X., Olson D.M., Fonarow G.C., Schwamm L.H., Smith E.E. (2012). Times from symptom onset to hospital arrival in the Get with the Guidelines—Stroke Program 2002 to 2009: Temporal trends and implications. Stroke.

[9] Saver J.L., Fonarow G.C., Smith E.E., Reeves M.J., Grau-Sepulveda M.V., Pan W., Olson D.M., Hernandez A.F., Peterson E.D., Schwamm L.H. (2013). Time to treatment with intravenous tissue plasminogen activator and outcome from acute ischemic stroke. JAMA.

[10] Khatri P., Abruzzo T., Yeatts S.D., Nichols C., Broderick J.P., Tomsick T.A. (2009). Good clinical outcome after ischemic stroke with successful revascularization is time-dependent. Neurology.

[11] Eissa A., Krass I., Levi C., Sturm J., Ibrahim R., Bajorek B. (2013). Understanding the reasons behind the low utilisation of thrombolysis in stroke. Australas. Med. J..

[12] Abdalkader M., Siegler J.E., Lee J.S., Yaghi S., Qiu Z., Huo X., Miao Z., Campbell B.C.V., Nguyen T.N. (2023). Neuroimaging of Acute Ischemic Stroke: Multimodal Imaging Approach for Acute Endovascular Therapy. J. Stroke.

[13] Psychogios K., Safouris A., Kargiotis O., Magoufis G., Andrikopoulou A., Papageorgiou E., Chondrogianni M., Papadimitropoulos G., Polyzogopoulou E., Spiliopoulos S. (2021). Advanced Neuroimaging Preceding Intravenous Thrombolysis in Acute Ischemic Stroke Patients Is Safe and Effective. J. Clin. Med..

[14] Magoufis G., Safouris A., Raphaeli G., Kargiotis O., Psychogios K., Krogias C., Palaiodimou L., Spiliopoulos S., Polizogopoulou E., Mantatzis M. (2021). Acute reperfusion therapies for acute ischemic stroke patients with unknown time of symptom onset or in extended time windows: An individualized approach. Ther. Adv. Neurol. Disord..

[15] Altersberger V.L., Sibolt G., Enz L.S., Hametner C., Scheitz J.F., Henon H., Bigliardi G., Strambo D., Martinez-Majander N., Stolze L.J. (2023). Intravenous Thrombolysis 4.5-9 Hours After Stroke Onset: A Cohort Study from the TRISP Collaboration. Ann. Neurol..

[16] Palaiodimou L., Katsanos A.H., Turc G., Romoli M., Theodorou A., Lemmens R., Sacco S., Velonakis G., Vlachopoulos C., Tsivgoulis G. (2024). Tenecteplase for the treatment of acute ischemic stroke in the extended time window: A systematic review and meta-analysis. Ther. Adv. Neurol. Disord..

[17] Albers G.W., Jumaa M., Purdon B., Zaidi S.F., Streib C., Shuaib A., Sangha N., Kim M., Froehler M.T., Schwartz N.E. (2024). Tenecteplase for Stroke at 4.5 to 24 Hours with Perfusion-Imaging Selection. N. Engl. J. Med..

[18] Xiong Y., Campbell B.C.V., Schwamm L.H., Meng X., Jin A., Parsons M.W., Fisher M., Jiang Y., Che F., Wang L. (2024). Tenecteplase for Ischemic Stroke at 4.5 to 24 Hours without Thrombectomy. N. Engl. J. Med..

[19] Yan S., Zhou Y., Lansberg M.G., Liebeskind D.S., Yuan C., Yu H., Chen F., Chen H., Zhang B., Mao L. (2025). Alteplase for Posterior Circulation Ischemic Stroke at 4.5 to 24 Hours. N. Engl. J. Med..

[20] Page M.J., McKenzie J.E., Bossuyt P.M., Boutron I., Hoffmann T.C., Mulrow C.D., Shamseer L., Tetzlaff J.M., Akl E.A., Brennan S.E. (2021). The PRISMA 2020 statement: An updated guideline for reporting systematic reviews. Bmj.

[21] Richardson W.S., Wilson M.C., Nishikawa J., Hayward R.S. (1995). The well-built clinical question: A key to evidence-based decisions. ACP J. Club.

[22] Sterne J.A.C., Savović J., Page M.J., Elbers R.G., Blencowe N.S., Boutron I., Cates C.J., Cheng H.Y., Corbett M.S., Eldridge S.M. (2019). RoB 2: A revised tool for assessing risk of bias in randomised trials. Bmj.

[23] Saver J.L., Chaisinanunkul N., Campbell B.C.V., Grotta J.C., Hill M.D., Khatri P., Landen J., Lansberg M.G., Venkatasubramanian C., Albers G.W. (2021). Standardized Nomenclature for Modified Rankin Scale Global Disability Outcomes: Consensus Recommendations from Stroke Therapy Academic Industry Roundtable XI. Stroke.

[24] Mendes D., Alves C., Batel-Marques F. (2017). Number needed to treat (NNT) in clinical literature: An appraisal. BMC Med..

[25] McGrath S., Zhao X., Steele R., Thombs B.D., Benedetti A. (2020). Estimating the sample mean and standard deviation from commonly reported quantiles in meta-analysis. Stat. Methods Med. Res..

[26] DerSimonian R., Laird N. (2015). Meta-analysis in clinical trials revisited. Contemp. Clin. Trials.

[27] Egger M., Davey Smith G., Schneider M., Minder C. (1997). Bias in meta-analysis detected by a simple, graphical test. Bmj.

[28] Balduzzi S., Rücker G., Schwarzer G. (2019). How to perform a meta-analysis with R: A practical tutorial. Evid. Based Ment. Health.

[29] Cheng X., Hong L., Lin L., Churilov L., Ling Y., Yang N., Fu J., Lu G., Yue Y., Zhang J. (2025). Tenecteplase Thrombolysis for Stroke up to 24 Hours After Onset with Perfusion Imaging Selection: The CHABLIS-T II Randomized Clinical Trial. Stroke.

[30] Emberson J., Lees K.R., Lyden P., Blackwell L., Albers G., Bluhmki E., Brott T., Cohen G., Davis S., Donnan G. (2014). Effect of treatment delay, age, and stroke severity on the effects of intravenous thrombolysis with alteplase for acute ischaemic stroke: A meta-analysis of individual patient data from randomised trials. Lancet.

[31] Lees K.R., Emberson J., Blackwell L., Bluhmki E., Davis S.M., Donnan G.A., Grotta J.C., Kaste M., von Kummer R., Lansberg M.G. (2016). Effects of Alteplase for Acute Stroke on the Distribution of Functional Outcomes: A Pooled Analysis of 9 Trials. Stroke.

[32] Coutts S.B., Ankolekar S., Appireddy R., Arenillas J.F., Assis Z., Bailey P., Barber P.A., Bazan R., Buck B.H., Butcher K.S. (2024). Tenecteplase versus standard of care for minor ischaemic stroke with proven occlusion (TEMPO-2): A randomised, open label, phase 3 superiority trial. Lancet.

[33] Sandercock P., Wardlaw J.M., Lindley R.I., Dennis M., Cohen G., Murray G., Innes K., Venables G., Czlonkowska A., Kobayashi A. (2012). The benefits and harms of intravenous thrombolysis with recombinant tissue plasminogen activator within 6 h of acute ischaemic stroke (the third international stroke trial [IST-3]): A randomised controlled trial. Lancet.

[34] Thomalla G., Boutitie F., Ma H., Koga M., Ringleb P., Schwamm L.H., Wu O., Bendszus M., Bladin C.F., Campbell B.C.V. (2020). Intravenous alteplase for stroke with unknown time of onset guided by advanced imaging: Systematic review and meta-analysis of individual patient data. Lancet.

[35] Chen H.S., Chen M.R., Cui Y., Shen X.Y., Zhang H., Lu J., Zhao L.W., Duan Y.J., Li J., Wang Y.M. (2025). Tenecteplase Plus Butyphthalide for Stroke Within 4.5–6 Hours of Onset (EXIT-BT): A Phase 2 Study. Transl. Stroke Res..

[36] Yogendrakumar V., Campbell B.C., Churilov L., Garcia-Esperon C., Choi P.M., Cordato D.J., Guha P., Sharma G., Chen C., McDonald A. (2025). Extending the time window for tenecteplase by effective reperfusion of penumbral tissue in patients with large vessel occlusion: Rationale and design of a multicenter, prospective, randomized, open-label, blinded-endpoint, controlled phase 3 trial. Int. J. Stroke.

[37] Luo Z., Zhou Y., He Y., Yan S., Chen Z., Zhang X., Chen Y., Tong L.S., Zhong W., Hu H. (2024). Treatment with intravenous alteplase in ischaemic stroke patients with onset time between 4.5 and 24 hours (HOPE): Protocol for a randomised, controlled, multicentre study. Stroke Vasc. Neurol..

[38] Ma H., Campbell B.C.V., Parsons M.W., Churilov L., Levi C.R., Hsu C., Kleinig T.J., Wijeratne T., Curtze S., Dewey H.M. (2019). Thrombolysis Guided by Perfusion Imaging up to 9 Hours after Onset of Stroke. N. Engl. J. Med..

[39] Ringleb P., Bendszus M., Bluhmki E., Donnan G., Eschenfelder C., Fatar M., Kessler C., Molina C., Leys D., Muddegowda G. (2019). Extending the time window for intravenous thrombolysis in acute ischemic stroke using magnetic resonance imaging-based patient selection. Int. J. Stroke.

[40] Davis S.M., Donnan G.A., Parsons M.W., Levi C., Butcher K.S., Peeters A., Barber P.A., Bladin C., De Silva D.A., Byrnes G. (2008). Effects of alteplase beyond 3 h after stroke in the Echoplanar Imaging Thrombolytic Evaluation Trial (EPITHET): A placebo-controlled randomised trial. Lancet Neurol..

[41] Campbell B.C.V., Ma H., Ringleb P.A., Parsons M.W., Churilov L., Bendszus M., Levi C.R., Hsu C., Kleinig T.J., Fatar M. (2019). Extending thrombolysis to 4·5–9 h and wake-up stroke using perfusion imaging: A systematic review and meta-analysis of individual patient data. Lancet.

[42] Koga M., Yamamoto H., Inoue M., Asakura K., Aoki J., Hamasaki T., Kanzawa T., Kondo R., Ohtaki M., Itabashi R. (2020). Thrombolysis with Alteplase at 0.6 mg/kg for Stroke with Unknown Time of Onset: A Randomized Controlled Trial. Stroke.

[43] Roaldsen M.B., Eltoft A., Wilsgaard T., Christensen H., Engelter S.T., Indredavik B., Jatužis D., Karelis G., Kõrv J., Lundström E. (2023). Safety and efficacy of tenecteplase in patients with wake-up stroke assessed by non-contrast CT (TWIST): A multicentre, open-label, randomised controlled trial. Lancet Neurol..

[44] Thomalla G., Simonsen C.Z., Boutitie F., Andersen G., Berthezene Y., Cheng B., Cheripelli B., Cho T.H., Fazekas F., Fiehler J. (2018). MRI-Guided Thrombolysis for Stroke with Unknown Time of Onset. N. Engl. J. Med..

[45] Wang L., Dai Y.J., Cui Y., Zhang H., Jiang C.H., Duan Y.J., Zhao Y., Feng Y.F., Geng S.M., Zhang Z.H. (2023). Intravenous Tenecteplase for Acute Ischemic Stroke Within 4.5–24 Hours of Onset (ROSE-TNK): A Phase 2, Randomized, Multicenter Study. J. Stroke.

[46] The NINDS t-PA Stroke Study Group (1997). Intracerebral hemorrhage after intravenous t-PA therapy for ischemic stroke. Stroke.

[47] Hacke W., Kaste M., Bluhmki E., Brozman M., Dávalos A., Guidetti D., Larrue V., Lees K.R., Medeghri Z., Machnig T. (2008). Thrombolysis with alteplase 3 to 4.5 hours after acute ischemic stroke. N. Engl. J. Med..

[48] Wahlgren N., Ahmed N., Dávalos A., Ford G.A., Grond M., Hacke W., Hennerici M.G., Kaste M., Kuelkens S., Larrue V. (2007). Thrombolysis with alteplase for acute ischaemic stroke in the Safe Implementation of Thrombolysis in Stroke-Monitoring Study (SITS-MOST): An observational study. Lancet.

[49] Tsivgoulis G., Katsanos A.H., Sandset E.C., Turc G., Nguyen T.N., Bivard A., Fischer U., Khatri P. (2023). Thrombolysis for acute ischaemic stroke: Current status and future perspectives. Lancet Neurol..

[50] Aladawi M., Abuawwad M.T., Taha M.J.J., Kozaa Y.A., Alrubasy W.A., Hamad A., Alhnidi F.A., Elfil M., Najdawi Z., Peng X. (2025). Tenecteplase Beyond 4.5 Hours in Acute Ischemic Stroke: A Systematic Review and Meta-Analysis of Randomized Clinical Trials. J. Stroke.

[51] Rousseau J.F., Weber J.M., Alhanti B., Saver J.L., Messé S.R., Schwamm L.H., Fonarow G.C., Sheth K.N., Smith E.E., Mullen M.T. (2025). Short-Term Safety and Effectiveness for Tenecteplase and Alteplase in Acute Ischemic Stroke. JAMA Netw. Open.

[52] Palaiodimou L., Tsivgoulis G. (2025). Transitioning to Intravenous Tenecteplase for the Treatment of Acute Ischemic Stroke. JAMA Netw. Open.

[53] Turc G., Tsivgoulis G., Audebert H.J., Boogaarts H., Bhogal P., De Marchis G.M., Fonseca A.C., Khatri P., Mazighi M., Pérez de la Ossa N. (2022). European Stroke Organisation—European Society for Minimally Invasive Neurological Therapy expedited recommendation on indication for intravenous thrombolysis before mechanical thrombectomy in patients with acute ischaemic stroke and anterior circulation large vessel occlusion. Eur. Stroke J..

[54] Qiu Z., Li F., Sang H., Yuan G., Xie D., Zhou K., Li M., Meng Z., Kong Z., Ruan Z. (2025). Intravenous Tenecteplase before Thrombectomy in Stroke. N. Engl. J. Med..

[55] Günkan A., Ferreira M.Y., Vilardo M., Scarcia L., Bocanegra-Becerra J.E., Cardoso L.J.C., Fabrini Paleare L.F., de Oliveira Almeida G., Semione G., Ferreira C. (2025). Thrombolysis for Ischemic Stroke Beyond the 4.5-Hour Window: A Meta-Analysis of Randomized Clinical Trials. Stroke.

[56] Al-Janabi O.M., Jazayeri S.B., Toruno M.A., Mahmood Y.M., Ghozy S., Yaghi S., Rabinstein A.A., Kallmes D.F. (2024). Safety and efficacy of intravenous thrombolytic therapy in the extended window up to 24 hours: A systematic review and meta-analysis. Ann. Clin. Transl. Neurol..

