# Supplementary material for: Efficacy and Safety of Intravenous Thrombolysis in the Extended Time Window for Acute Ischemic Stroke: A Systematic Review and Meta-Analysis
Source: J Clin Med. 2025 Aug 4;14(15):5474. doi: 10.3390/jcm14155474 (PMC12347962; doi:10.3390/jcm14155474)

Supplement:

eMethods

eTables S1-S2

eReferences

eFigures S1-S15

eMethods

Complete search algorithm used in MEDLINE (using PubMed).

("stroke"[tiab] OR "ischemic stroke"[tiab] OR "acute ischemic stroke"[tiab] OR "brain ischemia"[tiab])

AND

(thrombolysis[tiab] OR "intravenous thrombolysis"[tiab] OR IVT[tiab]

OR "tissue plasminogen activator"[tiab] OR rtPA[tiab] OR alteplase[tiab]

OR tenecteplase[tiab] OR TNK[tiab])

AND

("delayed treatment"[tiab] OR "extended window"[tiab] OR "late treatment"[tiab]

OR "treatment window"[tiab] OR "beyond 4.5 hours"[tiab] OR "after 4.5 hours"[tiab]

OR "late thrombolysis"[tiab] OR "late window"[tiab] OR "expanded window"[tiab])

Complete search algorithm used in Scopus.

(TITLE-ABS ("stroke" OR "ischemic stroke" OR "acute ischemic stroke" OR "brain ischemia"))

AND

(TITLE-ABS ("thrombolysis" OR "intravenous thrombolysis" OR "IVT" OR "tissue plasminogen activator" OR "rtPA" OR "alteplase" OR "tenecteplase" OR "TNK"))
AND

(TITLE-ABS ("beyond 4.5 hours" OR "after 4.5 hours" OR "late thrombolysis" OR "extended window" OR "late window" OR "late treatment" OR "expanded window" OR "delayed treatment" OR "treatment window"))

Complete search algorithm used in ClinicalTrials.gov.

Ischemic stroke AND (thrombolysis OR alteplase OR rtPA OR tenecteplase OR TNK) AND (beyond 4.5 hours OR after 4.5 hours OR late thrombolysis OR extended window OR late window)

eTable S1: Excluded studies with reasons of exclusion

Study name	Reason for exclusion
ETERNAL LVO 2025[1]	Wrong control population
EXIT-BT 2024[2]	Wrong study design
CHABLIS-T II 2025[3]	Wrong control population
Emberson et al. 2014[4]	Duplicate data with Cambell et al.
Lees et al. 2016[5]	Duplicate data with Cambell et al.
TEMPO-2 2024[6]	Wrong time frame
IST-3 2012[7]	Without outcomes of interest
Thomalla et al. 2020[8]	Duplicate data with Cambell et al.

eTable S2: Pooled proportion of outcomes of interest in each arm

Outcome	IVT + BMT<br>% (95%CI)	BMT<br>% (95%CI)
**Efficacy outcomes**		
Excellent functional outcome	46 (37-54)	37 (30-45)
Good functional outcome	61 (51-71)	51 (43-58)
Reduced disability	NA	NA
**Safety outcomes**		
Symptomatic intracranial hemorrhage	3 (2-4)	1 (0-1)
Any intracranial hemorrhage	12 (8-16)	9 (4-16)
All-cause mortality	9 (6-13)	7 (4-11)

IVT: Intravenous thrombolysis; BMT: Best medical treatment; CI: Confidence interval.

eReferences

1. Yogendrakumar V, Campbell BC, Churilov L, Garcia-Esperon C, Choi PM, Cordato DJ, et al. Extending the time window for tenecteplase by effective reperfusion of penumbral tissue in patients with large vessel occlusion: Rationale and design of a multicenter, prospective, randomized, open-label, blinded-endpoint, controlled phase 3 trial. Int J Stroke. 2025;20(3):367-72.
2. Chen HS, Chen MR, Cui Y, Shen XY, Zhang H, Lu J, et al. Tenecteplase Plus Butyphthalide for Stroke Within 4.5-6 Hours of Onset (EXIT-BT): a Phase 2 Study. Transl Stroke Res. 2025;16(3):575-83.
3. Cheng X, Hong L, Lin L, Churilov L, Ling Y, Yang N, et al. Tenecteplase Thrombolysis for Stroke up to 24 Hours After Onset With Perfusion Imaging Selection: The CHABLIS-T II Randomized Clinical Trial. Stroke. 2025;56(2):344-54.
4. Emberson J, Lees KR, Lyden P, Blackwell L, Albers G, Bluhmki E, et al. Effect of treatment delay, age, and stroke severity on the effects of intravenous thrombolysis with alteplase for acute ischaemic stroke: a meta-analysis of individual patient data from randomised trials. Lancet. 2014;384(9958):1929-35.
5. Lees KR, Emberson J, Blackwell L, Bluhmki E, Davis SM, Donnan GA, et al. Effects of Alteplase for Acute Stroke on the Distribution of Functional Outcomes: A Pooled Analysis of 9 Trials. Stroke. 2016;47(9):2373-9.
6. Coutts SB, Ankolekar S, Appireddy R, Arenillas JF, Assis Z, Bailey P, et al. Tenecteplase versus standard of care for minor ischaemic stroke with proven occlusion (TEMPO-2): a randomised, open label, phase 3 superiority trial. Lancet. 2024;403(10444):2597-605.
7. Sandercock P, Wardlaw JM, Lindley RI, Dennis M, Cohen G, Murray G, et al. The benefits and harms of intravenous thrombolysis with recombinant tissue plasminogen activator within 6 h of acute ischaemic stroke (the third international stroke trial [IST-3]): a randomised controlled trial. Lancet. 2012;379(9834):2352-63.
8. Thomalla G, Boutitie F, Ma H, Koga M, Ringleb P, Schwamm LH, et al. Intravenous alteplase for stroke with unknown time of onset guided by advanced imaging: systematic review and meta-analysis of individual patient data. Lancet. 2020;396(10262):1574-84.

eFigure S1. Forest plots presenting the mean age (in years) among patients treated with intravenous thrombolysis (IVT) together with best medical treatment (BMT; Panel A), the mean age (in years) among patients treated with BMT alone (Panel B), and the standardized mean difference of age (in years) among the patients treated with IVT together with BMT versus BMT alone (Panel C).

A.

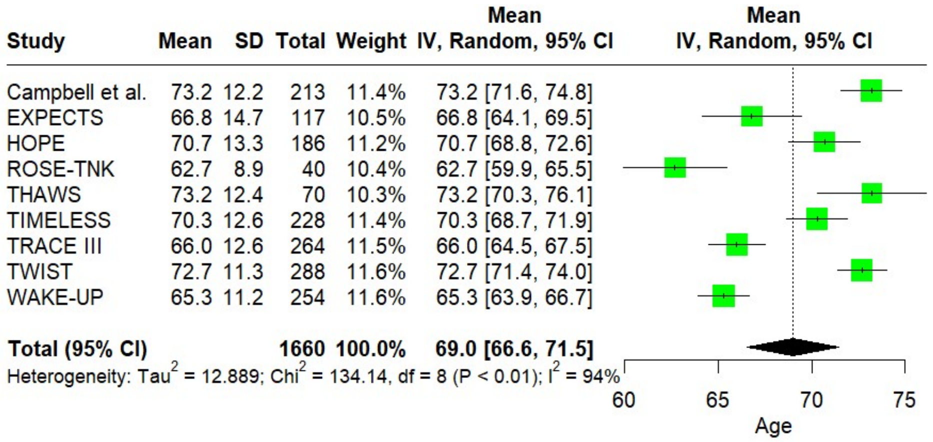

B.

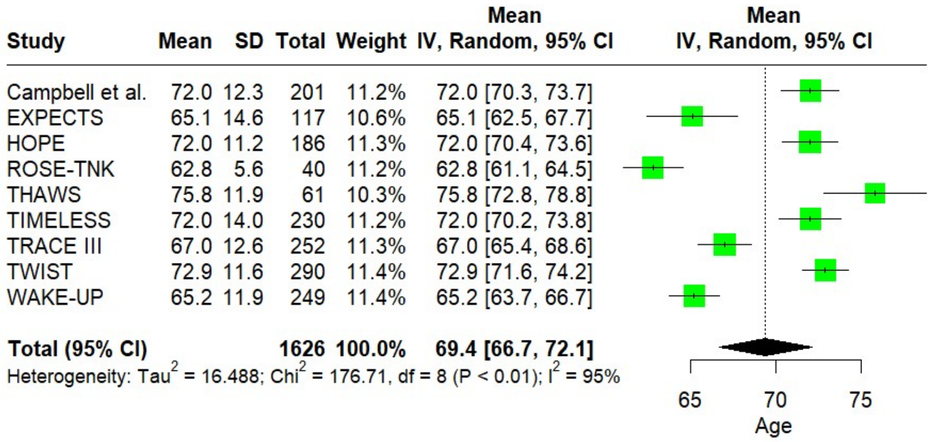

C.

Study	IVT Mean	IVT SD	IVT Total	BMT Mean	BMT SD	BMT Total	Weight	Std. Mean Difference IV, Random, 95% CI
Campbell et al.	73.2	12.2	213	72.0	12.3	201	12.6%	0.1 [-0.1, 0.3]
EXPECTS	66.8	14.7	117	65.1	14.6	117	7.1%	0.1 [-0.1, 0.4]
HOPE	70.7	13.3	186	72.0	11.2	186	11.3%	-0.1 [-0.3, 0.1]
ROSE-TNK	62.7	8.9	40	62.8	5.6	40	2.4%	-0.0 [-0.5, 0.4]
THAWS	73.2	12.4	70	75.8	11.9	61	4.0%	-0.2 [-0.6, 0.1]
TIMELESS	70.3	12.6	228	72.0	14.0	230	13.9%	-0.1 [-0.3, 0.1]
TRACE III	66.0	12.6	264	67.0	12.6	252	15.7%	-0.1 [-0.3, 0.1]
TWIST	72.7	11.3	288	72.9	11.6	290	17.6%	-0.0 [-0.2, 0.1]
WAKE-UP	65.3	11.2	254	65.2	11.9	249	15.3%	0.0 [-0.2, 0.2]
**Total (95% CI)**			**1660**			**1626**	**100.0%**	**-0.0 [-0.1, 0.0]**

Heterogeneity: $Tau^2 = 0$; $Chi^2 = 6.14$, df = 8 (P = 0.63); $I^2 = 0\%$
Test for overall effect: Z = -0.92 (P = 0.359)

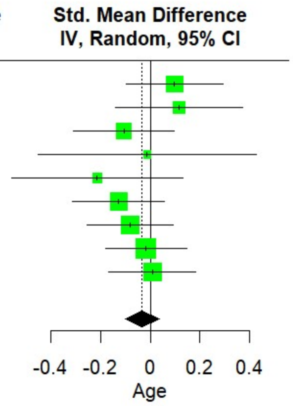

eFigure S2. Forest plots presenting the pooled proportion of female patients among those treated with IVT together with BMT (Panel A), the pooled proportion of female patients treated with BMT alone (Panel B), and the odds ratio of female patients treated with IVT together with BMT versus BMT (Panel C).

A.

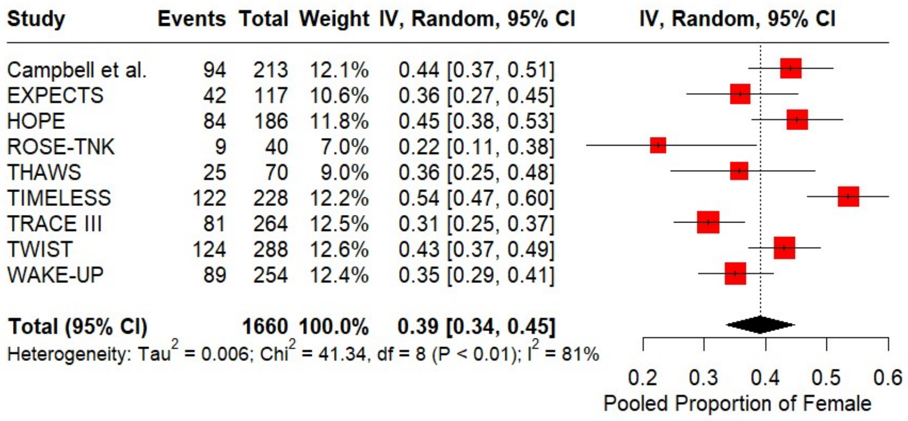

B.

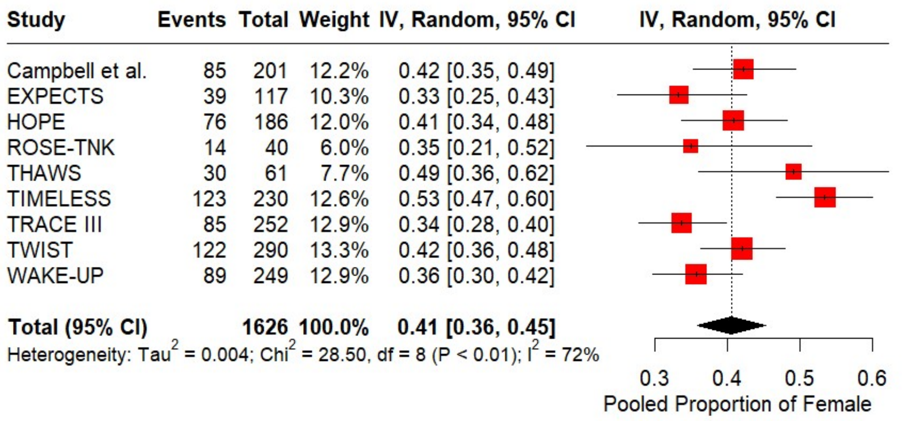

C.

Study	IVT Events	IVT Total	BMT Events	BMT Total	Weight	Odds Ratio IV, Random, 95% CI
Campbell et al.	94	213	85	201	13.1%	1.1 [0.7, 1.6]
EXPECTS	42	117	39	117	6.8%	1.1 [0.7, 1.9]
HOPE	84	186	76	186	11.7%	1.2 [0.8, 1.8]
ROSE-TNK	9	40	14	40	2.0%	0.5 [0.2, 1.4]
THAWS	25	70	30	61	4.0%	0.6 [0.3, 1.2]
TIMELESS	122	228	123	230	14.7%	1.0 [0.7, 1.4]
TRACE III	81	264	85	252	14.5%	0.9 [0.6, 1.3]
TWIST	124	288	122	290	18.2%	1.0 [0.7, 1.4]
WAKE-UP	89	254	89	249	14.8%	1.0 [0.7, 1.4]
**Total (95% CI)**		**1660**		**1626**	**100.0%**	**1.0 [0.9, 1.1]**

Heterogeneity: $Tau^2 = 0$; $Chi^2 = 5.53$, df = 8 (P = 0.70); $I^2 = 0\%$
Test for overall effect: Z = -0.20 (P = 0.844)

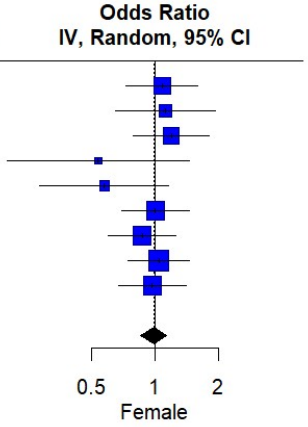

eFigure S3. Forest plots presenting the mean NIHSS among patients treated with IVT together with BMT (Panel A), the mean NIHSS among patients treated with BMT alone (Panel B), and the standardized mean difference of NIHSS among the patients treated with IVT together with BMT versus BMT alone (Panel C).

A.

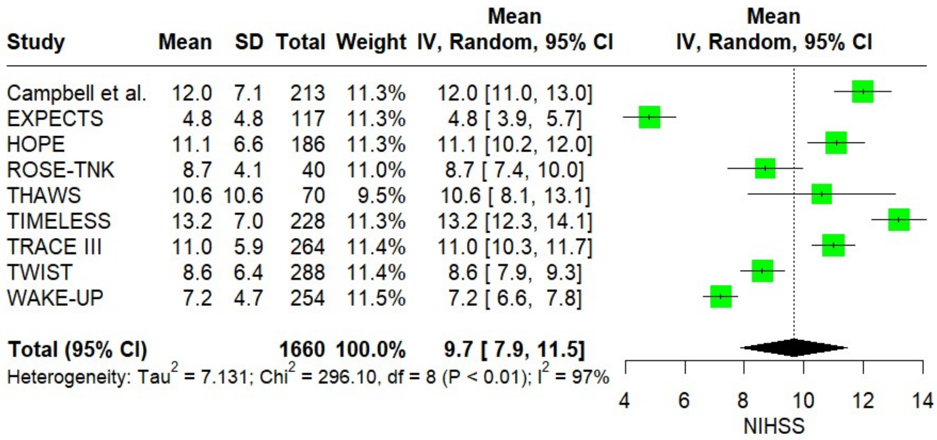

B.

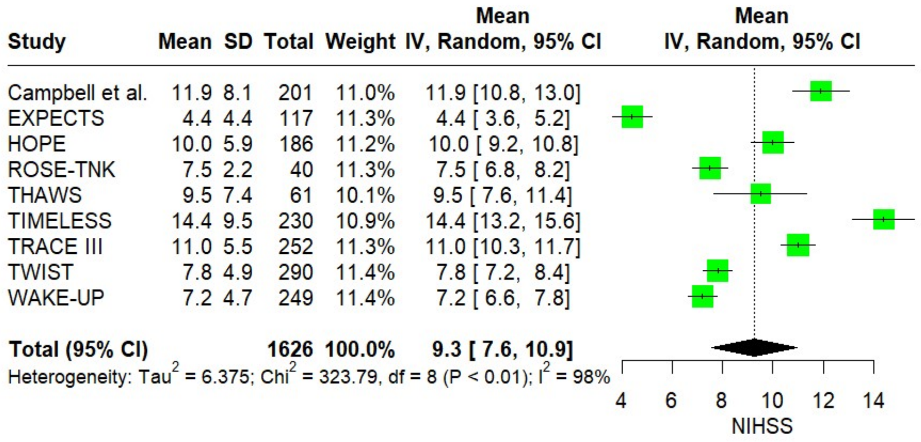

C.

Study	IVT Mean	IVT SD	IVT Total	BMT Mean	BMT SD	BMT Total	Weight	Std. Mean Difference IV, Random, 95% CI
Campbell et al.	12.0	7.1	213	11.9	8.1	201	12.7%	0.0 [-0.2, 0.2]
EXPECTS	4.8	4.8	117	4.4	4.4	117	7.9%	0.1 [-0.2, 0.3]
HOPE	11.1	6.6	186	10.0	5.9	186	11.7%	0.2 [-0.0, 0.4]
ROSE-TNK	8.7	4.1	40	7.5	2.2	40	2.9%	0.4 [-0.1, 0.8]
THAWS	10.6	10.6	70	9.5	7.4	61	4.7%	0.1 [-0.2, 0.5]
TIMELESS	13.2	7.0	228	14.4	9.5	230	13.8%	-0.1 [-0.3, 0.0]
TRACE III	11.0	5.9	264	11.0	5.5	252	15.1%	0.0 [-0.2, 0.2]
TWIST	8.6	6.4	288	7.8	4.9	290	16.4%	0.1 [-0.0, 0.3]
WAKE-UP	7.2	4.7	254	7.2	4.7	249	14.8%	0.0 [-0.2, 0.2]
**Total (95% CI)**			**1660**			**1626**	**100.0%**	**0.0 [-0.0, 0.1]**

Heterogeneity: $Tau^2 = 0.003$; $Chi^2 = 9.80$, df = 8 (P = 0.28); $I^2 = 18\%$
Test for overall effect: Z = 1.22 (P = 0.221)

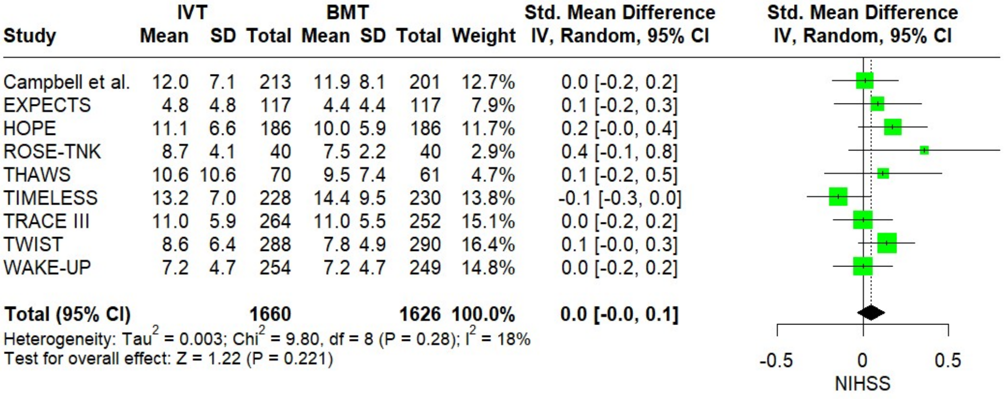

eFigure S4. Forest plots presenting the mean time since they were last know well (in hours) among patients treated with IVT together with BMT (Panel A), the mean time since they were last know well (in hours) among patients treated with BMT alone (Panel B), and the standardized mean difference of time (in hours) among the patients treated with IVT together with BMT versus BMT alone (Panel C).

A.

Study	Mean	SD	Total	Weight	Mean IV, Random, 95% CI
Campbell et al.	8.8	4.2	213	25.0%	8.8 [8.2, 9.4]
TIMELESS	12.6	4.9	228	24.8%	12.6 [12.0, 13.2]
TRACE III	12.7	5.4	264	24.8%	12.7 [12.0, 13.4]
WAKE-UP	10.1	2.8	254	25.4%	10.1 [9.8, 10.4]
**Total (95% CI)**			**959**	**100.0%**	**11.0 [9.3, 12.7]**

Heterogeneity: $Tau^2 = 2.956$; $Chi^2 = 125.13$, df = 3 ($P < 0.01$); $I^2 = 98\%$

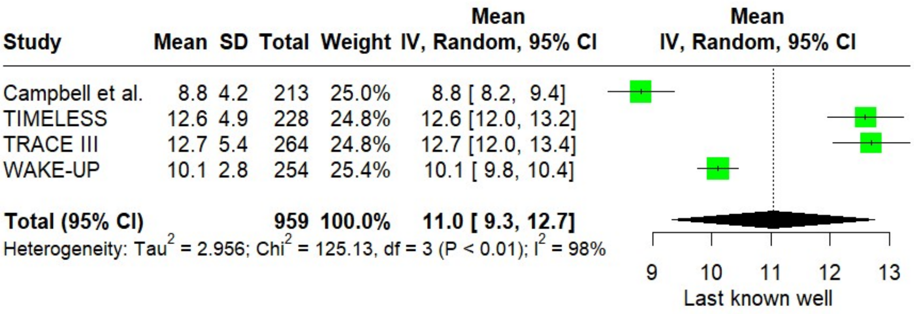

B.

Study	Mean	SD	Total	Weight	Mean IV, Random, 95% CI
Campbell et al.	8.9	4.2	201	25.1%	8.9 [8.3, 9.5]
TIMELESS	13.0	5.9	230	24.8%	13.0 [12.2, 13.8]
TRACE III	13.8	6.6	252	24.6%	13.8 [13.0, 14.6]
WAKE-UP	10.2	2.9	249	25.5%	10.2 [9.8, 10.6]
**Total (95% CI)**			**932**	**100.0%**	**11.5 [9.5, 13.4]**

Heterogeneity: $Tau^2 = 3.981$; $Chi^2 = 134.78$, df = 3 ($P < 0.01$); $I^2 = 98\%$

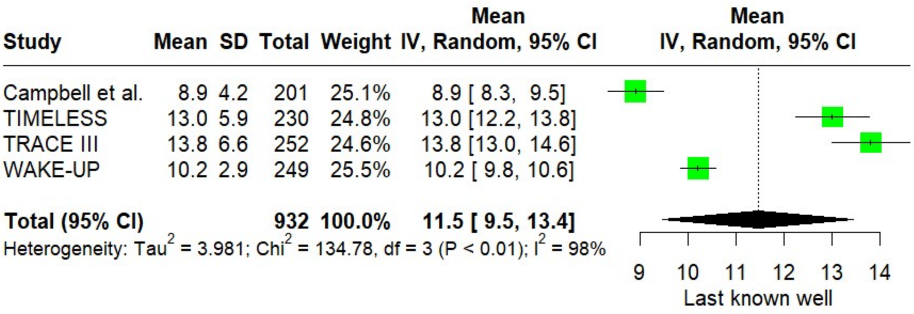

C.

Study	IVT Mean	IVT SD	IVT Total	BMT Mean	BMT SD	BMT Total	Weight	Std. Mean Difference IV, Random, 95% CI
Campbell et al.	8.8	4.2	213	8.9	4.2	201	21.9%	-0.0 [-0.2, 0.2]
TIMELESS	12.6	4.9	228	13.0	5.9	230	24.2%	-0.1 [-0.3, 0.1]
TRACE III	12.7	5.4	264	13.8	6.6	252	27.2%	-0.2 [-0.4, -0.0]
WAKE-UP	10.1	2.8	254	10.2	2.9	249	26.6%	-0.0 [-0.2, 0.1]
**Total (95% CI)**			**959**			**932**	**100.0%**	**-0.1 [-0.2, 0.0]**

Heterogeneity: $Tau^2 = 0$; $Chi^2 = 1.93$, df = 3 (P = 0.59); $I^2 = 0\%$
Test for overall effect: Z = -1.78 (P = 0.075)

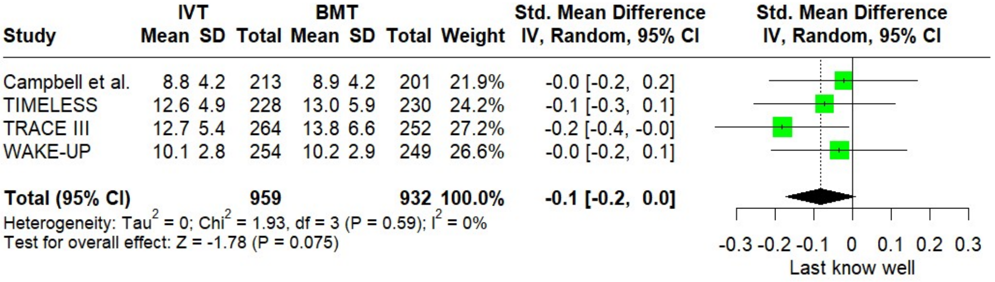

eFigure S5. Traffic light plot (A) and summary plot (B) presenting the quality assessment of the included randomized-controlled clinical trials (RCTs) and the individual patient-data meta-analysis (IPDM) of RCTs, using the Cochrane Collaboration tool (RoB 2).

A

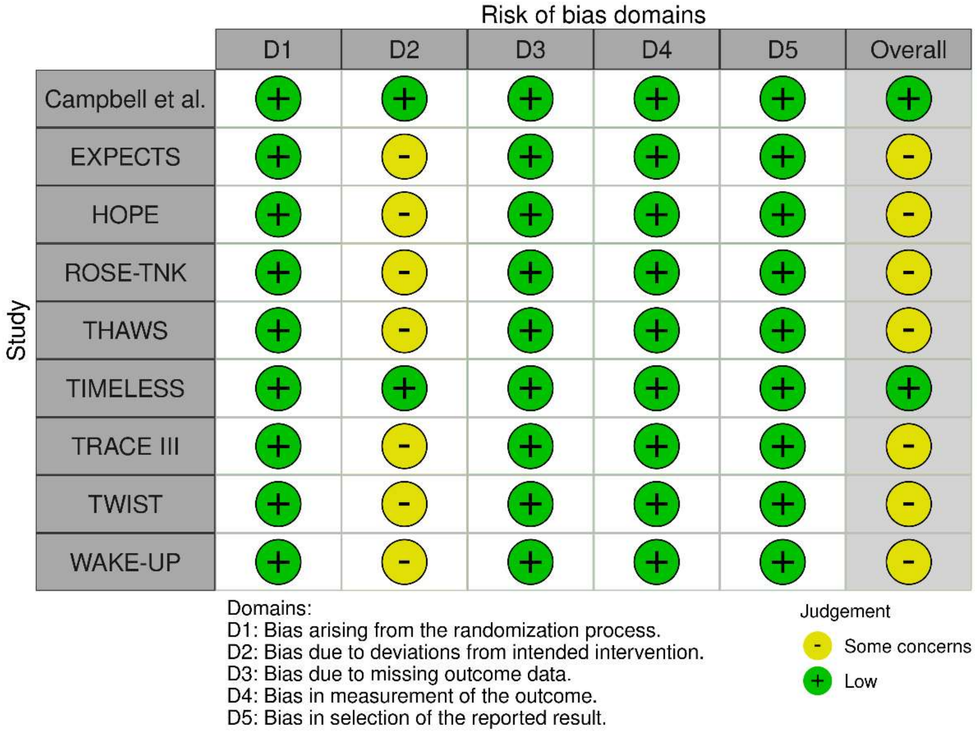

B

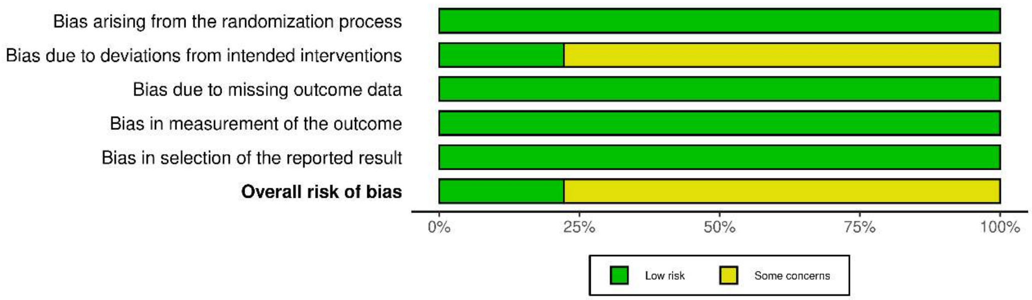

eFigure S6. Forest plot presenting the risk ratio of excellent functional outcome at 3 months among patients receiving IVT together with BMT versus BMT alone, stratified by thrombolytic agent used (Panel A), stratified by time of presentation (Panel B), stratified by neuroimaging used in each study (Panel C) and stratified by affected circulation (Panel D).

A

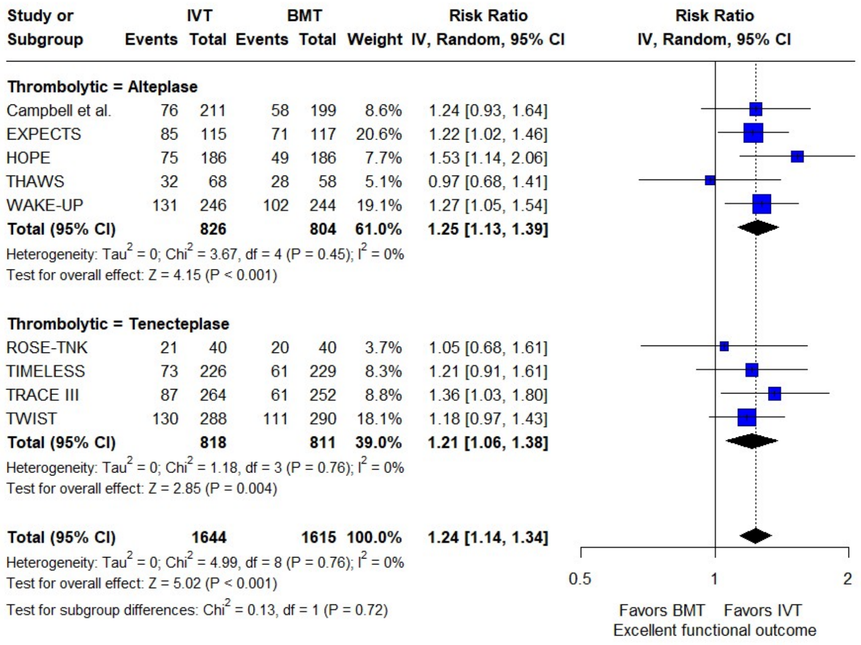

B

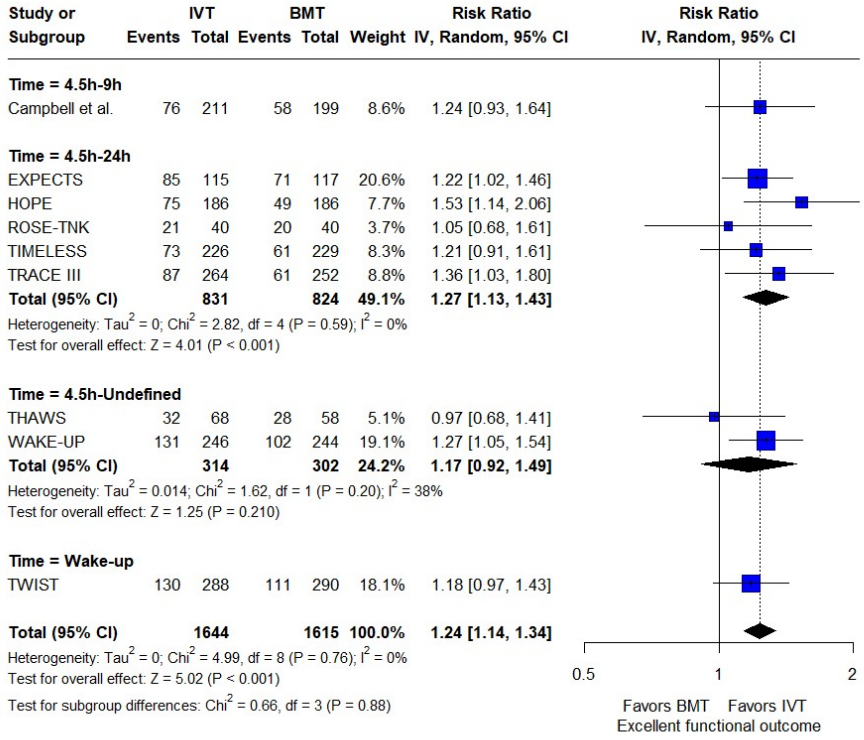

C

Study or Subgroup	IVT Events	IVT Total	BMT Events	BMT Total	Weight	Risk Ratio IV, Random, 95% CI
**Neuroimaging = Advanced**						
Campbell et al.	76	211	58	199	8.6%	1.24 [0.93, 1.64]
HOPE	75	186	49	186	7.7%	1.53 [1.14, 2.06]
ROSE-TNK	21	40	20	40	3.7%	1.05 [0.68, 1.61]
THAWS	32	68	28	58	5.1%	0.97 [0.68, 1.41]
TIMELESS	73	226	61	229	8.3%	1.21 [0.91, 1.61]
TRACE III	87	264	61	252	8.8%	1.36 [1.03, 1.80]
WAKE-UP	131	246	102	244	19.1%	1.27 [1.05, 1.54]
**Total (95% CI)**		**1241**		**1208**	**61.3%**	**1.26 [1.13, 1.40]**
Heterogeneity: $Tau^2 = 0$; $Chi^2 = 4.62$, df = 6 (P = 0.59); $I^2 = 0\%$						
Test for overall effect: Z = 4.28 (P < 0.001)						
**Neuroimaging = Standard**						
EXPECTS	85	115	71	117	20.6%	1.22 [1.02, 1.46]
TWIST	130	288	111	290	18.1%	1.18 [0.97, 1.43]
**Total (95% CI)**		**403**		**407**	**38.7%**	**1.20 [1.05, 1.37]**
Heterogeneity: $Tau^2 = 0$; $Chi^2 = 0.06$, df = 1 (P = 0.81); $I^2 = 0\%$						
Test for overall effect: Z = 2.69 (P = 0.007)						
**Total (95% CI)**		**1644**		**1615**	**100.0%**	**1.24 [1.14, 1.34]**
Heterogeneity: $Tau^2 = 0$; $Chi^2 = 4.99$, df = 8 (P = 0.76); $I^2 = 0\%$						
Test for overall effect: Z = 5.02 (P < 0.001)						
Test for subgroup differences: $Chi^2 = 0.31$, df = 1 (P = 0.58)						

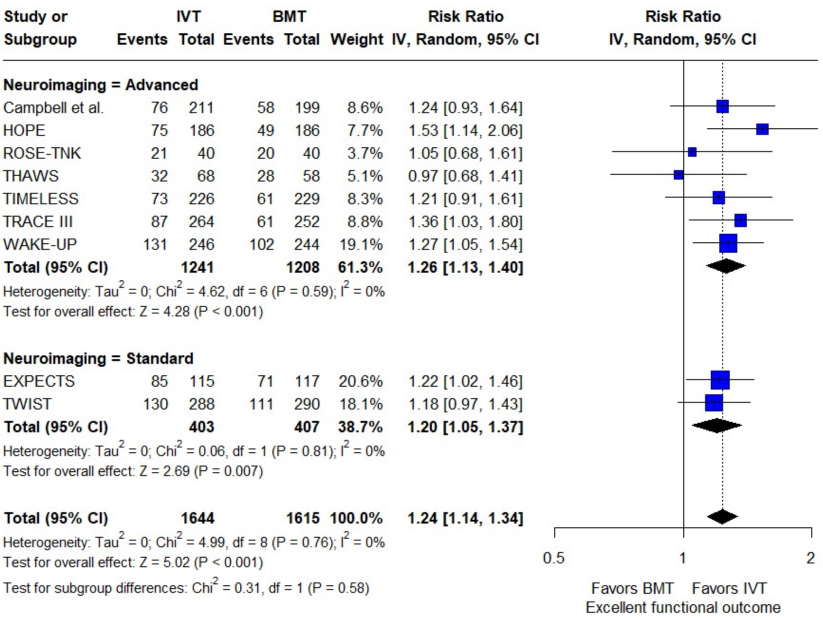

D

Study or Subgroup	IVT Events	IVT Total	BMT Events	BMT Total	Weight	Risk Ratio IV, Random, 95% CI
**Circulation = Anterior**						
Campbell et al.	76	211	58	199	8.6%	1.24 [0.93, 1.64]
TIMELESS	73	226	61	229	8.3%	1.21 [0.91, 1.61]
TRACE III	87	264	61	252	8.8%	1.36 [1.03, 1.80]
**Total (95% CI)**		**701**		**680**	**25.7%**	**1.27 [1.08, 1.49]**
Heterogeneity: $Tau^2 = 0$; $Chi^2 = 0.38$, df = 2 (P = 0.83); $I^2 = 0\%$						
Test for overall effect: Z = 2.88 (P = 0.004)						
**Circulation = Posterior**						
EXPECTS	85	115	71	117	20.6%	1.22 [1.02, 1.46]
**Circulation = Anterior & Posterior**						
HOPE	75	186	49	186	7.7%	1.53 [1.14, 2.06]
ROSE-TNK	21	40	20	40	3.7%	1.05 [0.68, 1.61]
THAWS	32	68	28	58	5.1%	0.97 [0.68, 1.41]
TWIST	130	288	111	290	18.1%	1.18 [0.97, 1.43]
WAKE-UP	131	246	102	244	19.1%	1.27 [1.05, 1.54]
**Total (95% CI)**		**828**		**818**	**53.7%**	**1.22 [1.08, 1.38]**
Heterogeneity: $Tau^2 = 0.002$; $Chi^2 = 4.46$, df = 4 (P = 0.35); $I^2 = 10\%$						
Test for overall effect: Z = 3.26 (P = 0.001)						
**Total (95% CI)**		**1644**		**1615**	**100.0%**	**1.24 [1.14, 1.34]**
Heterogeneity: $Tau^2 = 0$; $Chi^2 = 4.99$, df = 8 (P = 0.76); $I^2 = 0\%$						
Test for overall effect: Z = 5.02 (P < 0.001)						
Test for subgroup differences: $Chi^2 = 0.15$, df = 2 (P = 0.93)						

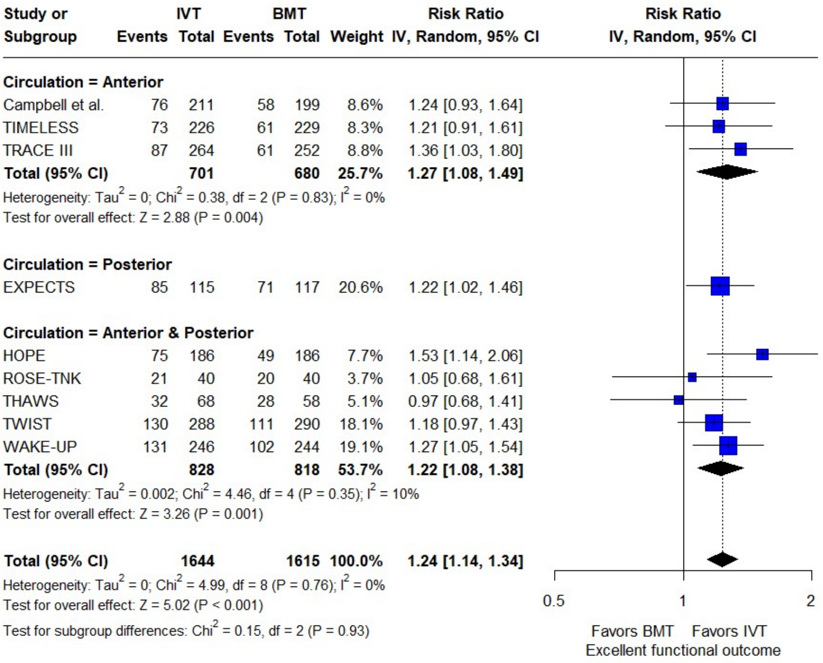

eFigure S7. Forest plot presenting the risk ratio of symptomatic intracranial hemorrhage (sICH) among patients receiving IVT together with BMT versus BMT alone, stratified by thrombolytic agent used (Panel A), stratified by time of presentation (Panel B), stratified by neuroimaging used in each study (Panel C) and stratified by affected circulation (Panel D).

A

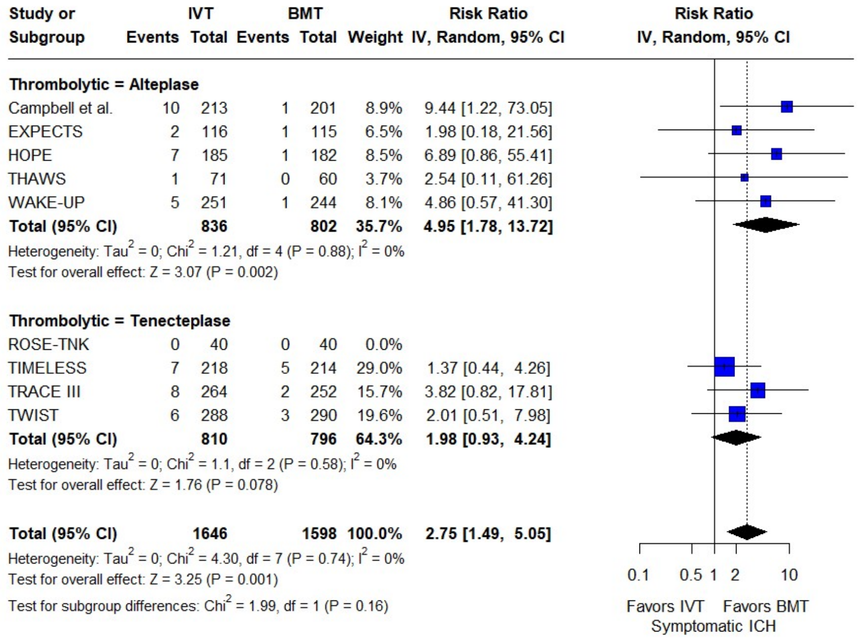

B

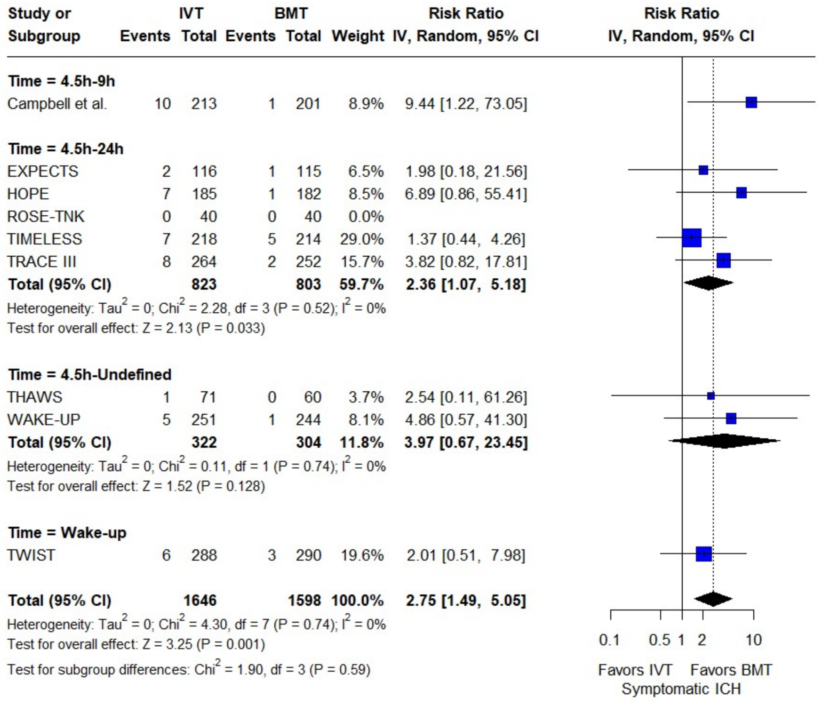

C

Study or Subgroup	IVT Events	IVT Total	BMT Events	BMT Total	Weight	Risk Ratio IV, Random, 95% CI
**Neuroimaging = Advanced**						
Campbell et al.	10	213	1	201	8.9%	9.44 [1.22, 73.05]
HOPE	7	185	1	182	8.5%	6.89 [0.86, 55.41]
ROSE-TNK	0	40	0	40	0.0%	
THAWS	1	71	0	60	3.7%	2.54 [0.11, 61.26]
TIMELESS	7	218	5	214	29.0%	1.37 [0.44, 4.26]
TRACE III	8	264	2	252	15.7%	3.82 [0.82, 17.81]
WAKE-UP	5	251	1	244	8.1%	4.86 [0.57, 41.30]
**Total (95% CI)**		**1242**		**1193**	**73.9%**	**3.07 [1.51, 6.24]**
Heterogeneity: $Tau^2 = 0$; $Chi^2 = 3.94$, df = 5 (P = 0.56); $I^2 = 0\%$						
Test for overall effect: Z = 3.10 (P = 0.002)						
**Neuroimaging = Standard**						
EXPECTS	2	116	1	115	6.5%	1.98 [0.18, 21.56]
TWIST	6	288	3	290	19.6%	2.01 [0.51, 7.98]
**Total (95% CI)**		**404**		**405**	**26.1%**	**2.01 [0.61, 6.61]**
Heterogeneity: $Tau^2 = 0$; $Chi^2 = 0$, df = 1 (P = 0.99); $I^2 = 0\%$						
Test for overall effect: Z = 1.14 (P = 0.252)						
**Total (95% CI)**		**1646**		**1598**	**100.0%**	**2.75 [1.49, 5.05]**
Heterogeneity: $Tau^2 = 0$; $Chi^2 = 4.30$, df = 7 (P = 0.74); $I^2 = 0\%$						
Test for overall effect: Z = 3.25 (P = 0.001)						
Test for subgroup differences: $Chi^2 = 0.36$, df = 1 (P = 0.55)						

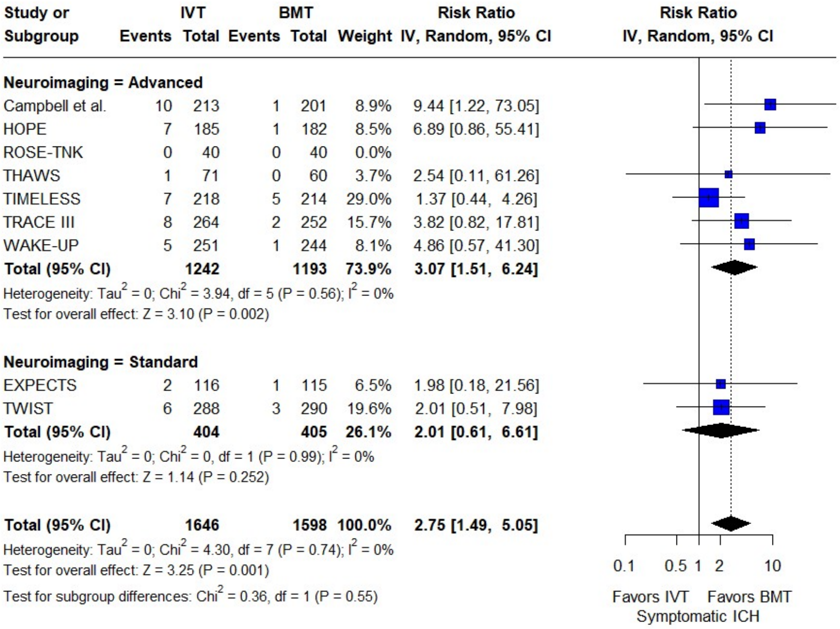

D

Study or Subgroup	IVT Events	IVT Total	BMT Events	BMT Total	Weight	Risk Ratio IV, Random, 95% CI
**Circulation = Anterior**						
Campbell et al.	10	213	1	201	8.9%	9.44 [1.22, 73.05]
TIMELESS	7	218	5	214	29.0%	1.37 [0.44, 4.26]
TRACE III	8	264	2	252	15.7%	3.82 [0.82, 17.81]
**Total (95% CI)**		**695**		**667**	**53.5%**	**2.87 [0.99, 8.32]**
Heterogeneity: $Tau^2 = 0.298$; $Chi^2 = 2.98$, df = 2 (P = 0.23); $I^2 = 33\%$						
Test for overall effect: Z = 1.94 (P = 0.052)						
**Circulation = Posterior**						
EXPECTS	2	116	1	115	6.5%	1.98 [0.18, 21.56]
**Circulation = Anterior & Posterior**						
HOPE	7	185	1	182	8.5%	6.89 [0.86, 55.41]
ROSE-TNK	0	40	0	40	0.0%	
THAWS	1	71	0	60	3.7%	2.54 [0.11, 61.26]
TWIST	6	288	3	290	19.6%	2.01 [0.51, 7.98]
WAKE-UP	5	251	1	244	8.1%	4.86 [0.57, 41.30]
**Total (95% CI)**		**835**		**816**	**39.9%**	**3.20 [1.22, 8.40]**
Heterogeneity: $Tau^2 = 0$; $Chi^2 = 1.12$, df = 3 (P = 0.77); $I^2 = 0\%$						
Test for overall effect: Z = 2.36 (P = 0.018)						
**Total (95% CI)**		**1646**		**1598**	**100.0%**	**2.75 [1.49, 5.05]**
Heterogeneity: $Tau^2 = 0$; $Chi^2 = 4.30$, df = 7 (P = 0.74); $I^2 = 0\%$						
Test for overall effect: Z = 3.25 (P = 0.001)						
Test for subgroup differences: $Chi^2 = 0.14$, df = 2 (P = 0.93)						

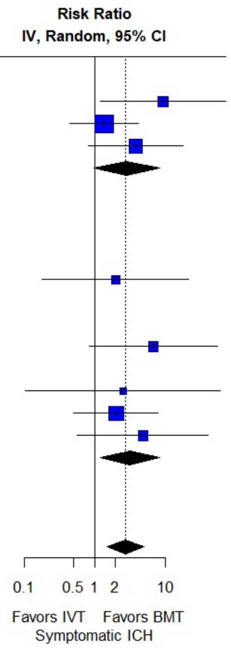

eFigure S8. Forest plot presenting the risk ratio of excellent functional outcome among patients receiving IVT together with BMT versus BMT alone, after excluding studies where patients underwent endovascular treatment (sensitivity analysis).

Study	IVT Events	IVT Total	BMT Events	BMT Total	Weight	Risk Ratio IV, Random, 95% CI
Campbell et al.	76	211	58	199	11.7%	1.24 [0.93, 1.64]
EXPECTS	85	115	71	117	28.0%	1.22 [1.02, 1.46]
HOPE	75	186	49	186	10.5%	1.53 [1.14, 2.06]
ROSE-TNK	21	40	20	40	5.1%	1.05 [0.68, 1.61]
THAWS	32	68	28	58	6.9%	0.97 [0.68, 1.41]
TRACE III	87	264	61	252	12.0%	1.36 [1.03, 1.80]
WAKE-UP	131	246	102	244	26.0%	1.27 [1.05, 1.54]
**Total (95% CI)**		**1130**		**1096**	**100.0%**	**1.25 [1.14, 1.38]**

Heterogeneity: $Tau^2 = 0$; $Chi^2 = 4.67$, df = 6 (P = 0.59); $I^2 = 0\%$

Test for overall effect: Z = 4.58 (P < 0.01)

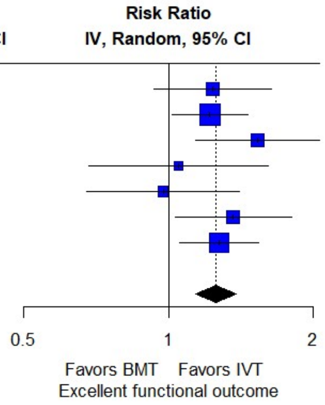

eFigure S9. Forest plot presenting the risk ratio of symptomatic intracranial hemorrhage among patients receiving IVT together with BMT versus BMT alone, after excluding studies where patients underwent endovascular treatment (sensitivity analysis).

Study	IVT Events	IVT Total	BMT Events	BMT Total	Weight	Risk Ratio IV, Random, 95% CI
Campbell et al.	10	213	1	201	17.3%	9.44 [1.22, 73.05]
EXPECTS	2	116	1	115	12.7%	1.98 [0.18, 21.56]
HOPE	7	185	1	182	16.6%	6.89 [0.86, 55.41]
ROSE-TNK	0	40	0	40	0.0%	
THAWS	1	71	0	60	7.1%	2.54 [0.11, 61.26]
TRACE III	8	264	2	252	30.5%	3.82 [0.82, 17.81]
WAKE-UP	5	251	1	244	15.8%	4.86 [0.57, 41.30]
**Total (95% CI)**		**1140**		**1094**	**100.0%**	**4.57 [1.95, 10.70]**

Heterogeneity: $Tau^2 = 0$; $Chi^2 = 1.29$, df = 5 ($P = 0.94$); $I^2 = 0\%$
Test for overall effect: $Z = 3.50$ ($P < 0.001$)

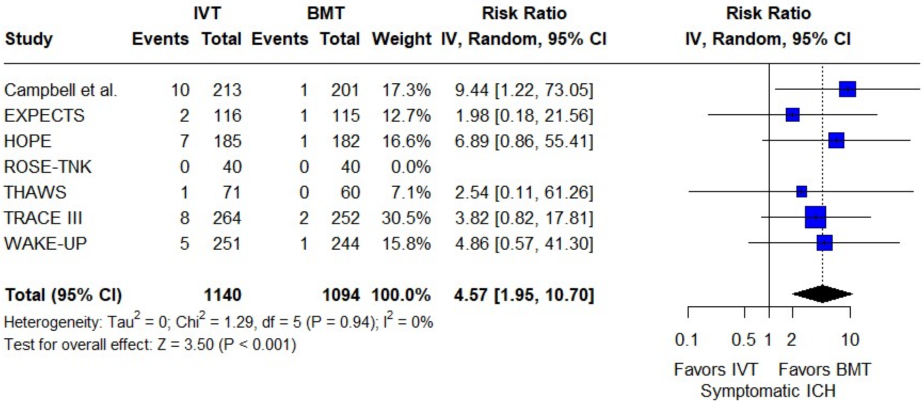

eFigure S10. Funnel plot on the reported rates of excellent functional outcome at 3 months (p for Egger's test= 0.62).

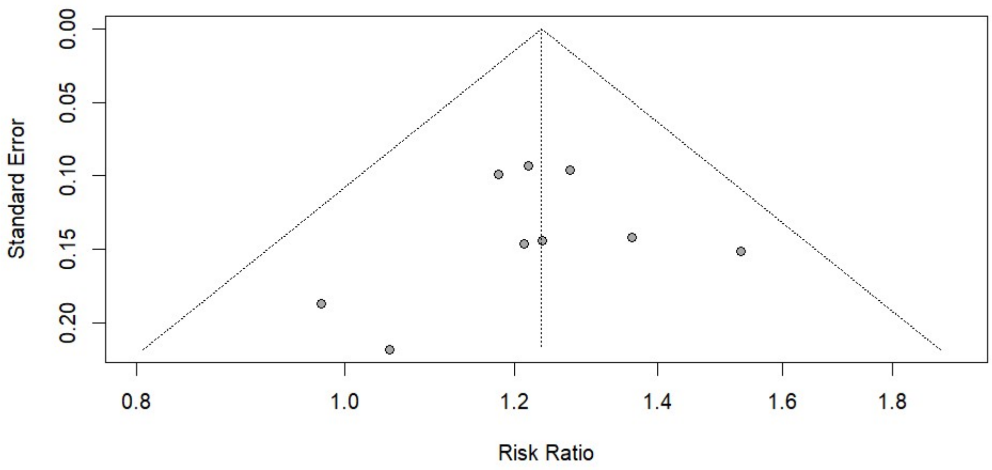

eFigure S11. Funnel plot on the reported rates of good functional outcome at 3 months (p for Egger's test= 0.73).

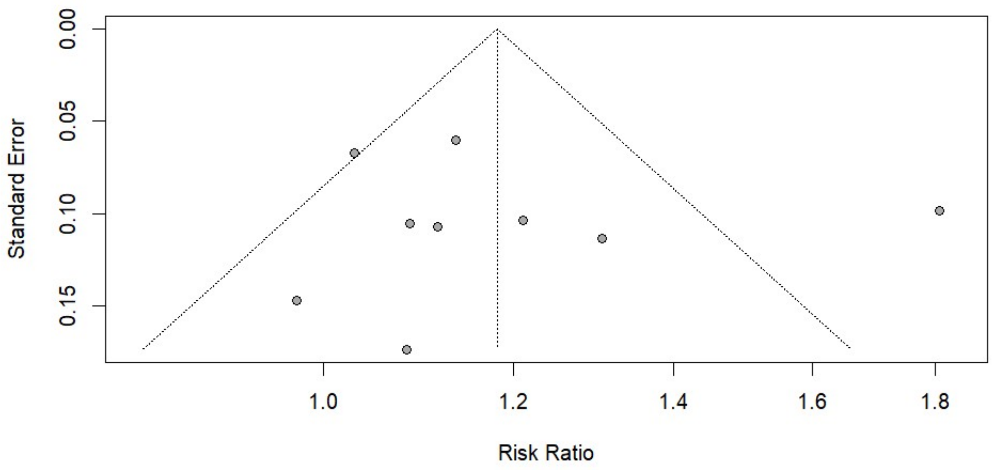

eFigure S12. Funnel plot on the reported odds of reduced disability at 3 months (p for Egger's test= 0.93).

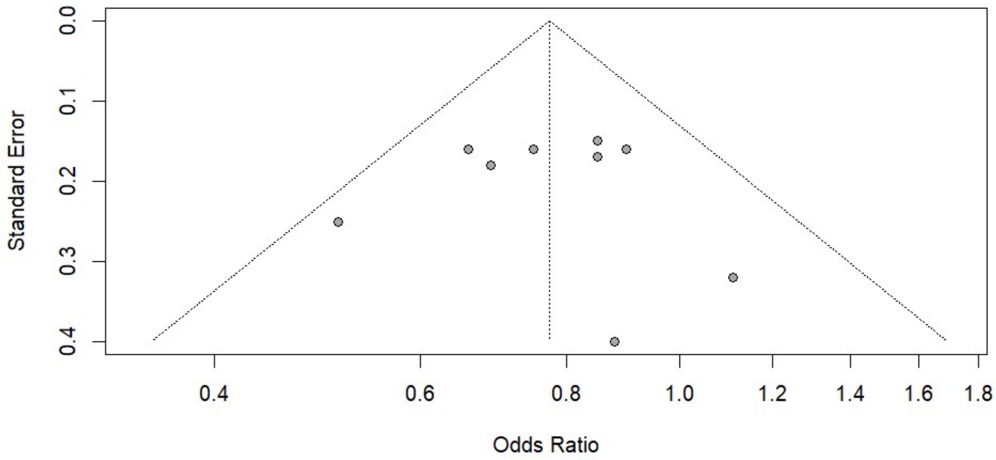

eFigure S13. Funnel plot on the reported rates of symptomatic intracranial hemorrhage (p for Egger's test= 0.13).

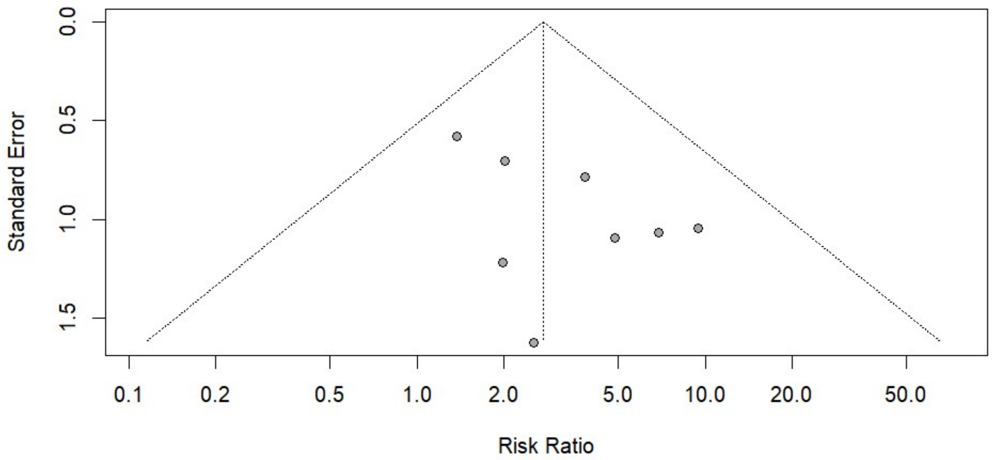

eFigure S14. Funnel plot on the reported rates of all-cause mortality at 3 months (p for Egger's test= 0.35).

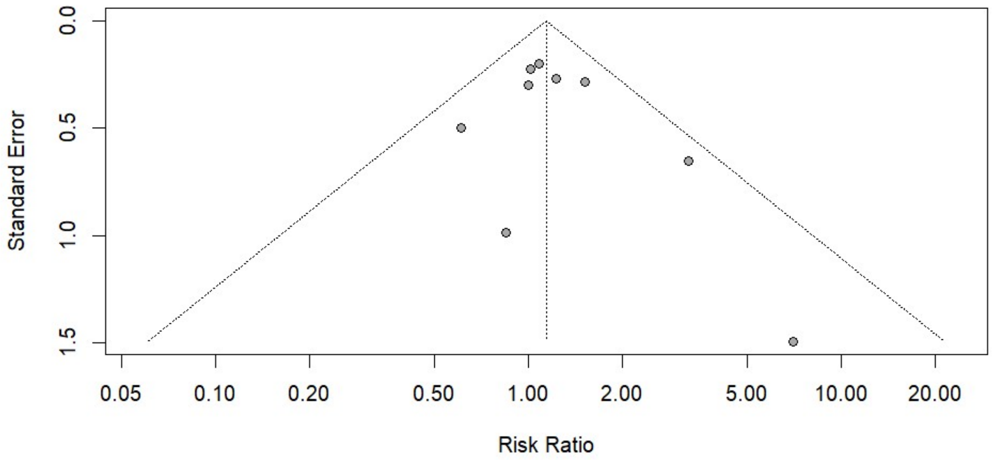

eFigure S15. Forest plot presenting the risk ratio of excellent functional outcome among patients receiving IVT together with BMT versus BMT alone, after excluding EXPECTS study (Panel A) and the risk ratio of good functional outcome among patients receiving IVT together with BMT versus BMT alone, after excluding EXPECTS study (Panel B).

A

Study	IVT Events	IVT Total	BMT Events	BMT Total	Weight	Risk Ratio IV, Random, 95% CI
Campbell et al.	76	211	58	199	10.8%	1.24 [0.93, 1.64]
HOPE	75	186	49	186	9.7%	1.53 [1.14, 2.06]
ROSE-TNK	21	40	20	40	4.7%	1.05 [0.68, 1.61]
THAWS	32	68	28	58	6.4%	0.97 [0.68, 1.41]
TIMELESS	73	226	61	229	10.5%	1.21 [0.91, 1.61]
TRACE III	87	264	61	252	11.1%	1.36 [1.03, 1.80]
TWIST	130	288	111	290	22.8%	1.18 [0.97, 1.43]
WAKE-UP	131	246	102	244	24.1%	1.27 [1.05, 1.54]
**Total (95% CI)**		**1529**		**1498**	**100.0%**	**1.24 [1.13, 1.36]**

Heterogeneity: $Tau^2 = 0$; $Chi^2 = 4.96$, df = 7 (P = 0.67); $I^2 = 0\%$
Test for overall effect: Z = 4.55 (P < 0.01)

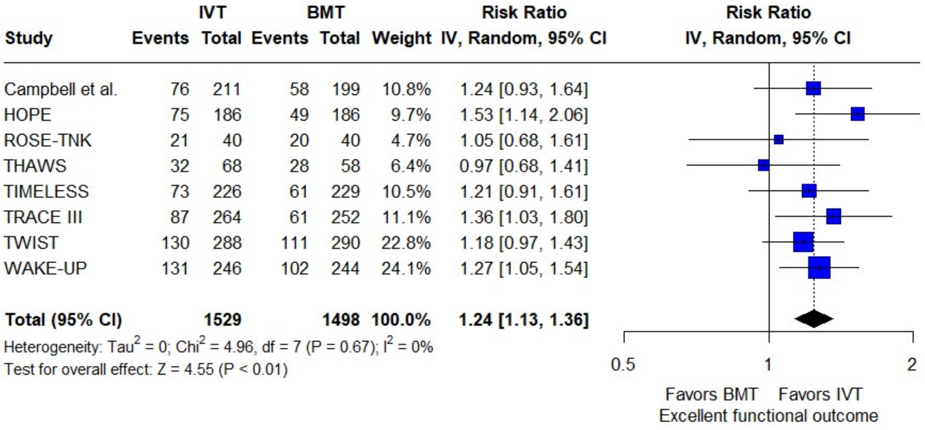

B

Study	IVT Events	IVT Total	BMT Events	BMT Total	Weight	Risk Ratio IV, Random, 95% CI
Campbell et al.	103	211	87	199	9.4%	1.12 [0.91, 1.38]
HOPE	103	186	85	186	10.1%	1.21 [0.99, 1.48]
ROSE-TNK	26	40	24	40	3.6%	1.08 [0.77, 1.52]
THAWS	40	68	35	58	5.0%	0.97 [0.73, 1.30]
TIMELESS	104	226	97	229	9.7%	1.09 [0.88, 1.34]
TRACE III	115	264	84	252	8.4%	1.31 [1.05, 1.63]
TWIST	177	288	173	290	24.0%	1.03 [0.90, 1.18]
WAKE-UP	182	246	159	244	29.8%	1.14 [1.01, 1.28]
**Total (95% CI)**		**1529**		**1498**	**100.0%**	**1.11 [1.04, 1.19]**

Heterogeneity: $Tau^2 = 0$; $Chi^2 = 5.00$, df = 7 (P = 0.66); $I^2 = 0\%$
Test for overall effect: Z = 3.25 (P < 0.01)

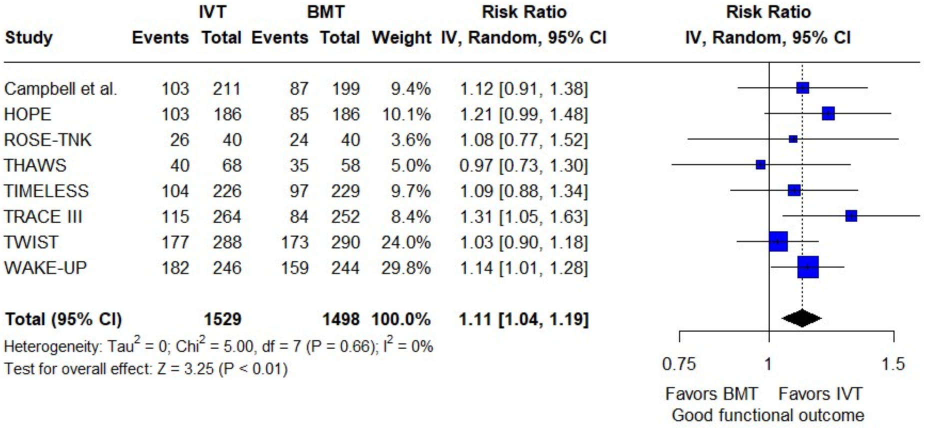

Supplement: Supplementary file 1 [file jcm-14-05474-s001.zip › jcm-3739801-supplementary.pdf]
